# Decoding Antioxidant and Antibacterial Potentials of Malaysian Green Seaweeds: *Caulerpa racemosa* and *Caulerpa lentillifera*

**DOI:** 10.3390/antibiotics8030152

**Published:** 2019-09-17

**Authors:** Wing-Fai Yap, Vangene Tay, Sie-Hui Tan, Yoon-Yen Yow, Jactty Chew

**Affiliations:** Department of Biological Sciences, Sunway University, Bandar Sunway, Selangor 47500, Malaysia; 15050396@imail.sunway.edu.my (W.-F.Y.); 13089883@imail.sunway.edu.my (V.T.); tsiehui@yahoo.com (S.-H.T.)

**Keywords:** antibacterial, antioxidant, macroalgae, *Caulerpa racemosa*, *Caulerpa lentillifera*, bioactive compounds, liquid chromatography–mass spectrometry

## Abstract

Seaweeds are gaining a considerable amount of attention for their antioxidant and antibacterial properties. *Caulerpa racemosa* and *Caulerpa lentillifera*, also known as ‘sea grapes’, are green seaweeds commonly found in different parts of the world, but the antioxidant and antibacterial potentials of Malaysian *C. racemosa* and *C. lentillifera* have not been thoroughly explored. In this study, crude extracts of the seaweeds were prepared using chloroform, methanol, and water. Total phenolic content (TPC) and total flavonoid content (TFC) were measured, followed by in vitro antioxidant activity determination using 2,2-diphenyl-1-picrylhydrazyl (DPPH) radical scavenging assay. Antibacterial activities of these extracts were tested against Methicillin-resistant *Staphylococcus aureus* (MRSA) and neuropathogenic *Escherichia coli* K1. Liquid chromatography–mass spectrometry (LCMS) analysis was then used to determine the possible compounds present in the extract with the most potent antioxidant and antibacterial activity. Results showed that *C. racemosa* chloroform extract had the highest TPC (13.41 ± 0.86 mg GAE/g), antioxidant effect (EC_50_ at 0.65 ± 0.03 mg/mL), and the strongest antibacterial effect (97.7 ± 0.30%) against MRSA. LCMS analysis proposed that the chloroform extracts of *C. racemosa* are mainly polyunsaturated and monounsaturated fatty acids, terpenes, and alkaloids. In conclusion, *C. racemosa* can be a great source of novel antioxidant and antibacterial agents, but isolation and purification of the bioactive compounds are needed to study their mechanism of action.

## 1. Introduction

Marine organisms such as seaweeds are a rich source of natural bioactive compounds. Studies have shown that seaweeds have various bioactive constituents such as proteins, polysaccharides, and secondary metabolites [1,2,3,4]. The presence of the bioactive compounds has been found to contribute to anti-inflammatory, antioxidant, and antimicrobial properties [5,6,7,8]. One of the bioactive compounds isolated from *Chondrus cripus*, iota-carrageenan, is currently used as an active ingredient in nasal spray products as part of the treatment for the common cold in adults. This sulphated polysaccharide binds to respiratory viruses, limiting the viral attachment to host cells [9]. Alginate, a polysaccharide isolated from brown seaweed (*Phaeophycae*), is formulated as an alginate-antacid to reduce postprandial acid reflux in patients with gastroesophageal reflux disease. The alginate-based formulation forms a layer on top of the gastric content, neutralizing the acid film [10]. In both cases, these bioactive compounds have demonstrated to be effective and beneficial, highlighting the invaluable pharmaceutical potential that seaweeds can offer. 

In recent years, the consumption of antioxidant rich foods has become a trend in view of their protective effects against reactive oxygen species (ROS) and the associated health conditions [11,12,13]. ROS are known to induce oxidative injury in human cells, leading to the onset of various chronic diseases, including Alzheimer’s disease, cancers, and aging [14,15,16,17,18]. Studies have shown that seaweeds possess bioactive compounds with strong antioxidant capacity to protect seaweeds against ROS [19,20,21]. The antioxidant compounds from seaweeds may belong to the main classes of phytochemicals known as phenolics and flavonoids (a derivative of phenolics), due to the presence of hydroxyl groups, which act as hydrogen-donors to stabilize the free radicals and to terminate the generation of new free radicals [22]. There are studies showing a positive correlation between total phenolic content and total flavonoid content in seaweed with antioxidant activity [23,24,25,26,27]. However, more in-depth studies are required in this area to identify the antioxidant compounds.

Additionally, seawater is teeming with microorganisms. A study found a significant degree of microbial diversity from ocean water samples, with 247 bacterial strains identified [28]. Interestingly, seaweeds can grow abundantly in seawater, indicating that they may have a plethora of bioactive compounds that allow their co-existence with microorganisms [29,30]. In fact, various studies have shown that seaweeds are a potent source of antibacterial agents [1,31]. The antibacterial potential has become an inspiration to researchers to develop effective antibacterial drugs to combat infectious diseases, which are claiming lives at an alarming rate. Each year, 50,000 deaths in the U.S. and Europe are attributed to antibiotic-resistant bacteria (ARB) diseases and if the problem with ARB remains unmonitored, it is estimated that they may lead to 10 million cases of death annually by 2050 [32]. The existence of ARB limits the types of antibiotics that can be prescribed to the patients and, thus, increases the mortality rate significantly. This scenario shows that bacteria are evolving and adapting quickly to newly-introduced antibiotics, while the development of novel antibiotics is still struggling to catch up.

In Malaysia, there are more than 386 taxa of marine seaweeds found, and many of these Malaysian seaweeds have been demonstrated to possess promising antibacterial, antioxidant, and antiviral properties [33,34,35,36,37]. *Caulerpa racemosa* and *Caulerpa lentillifera,* also known as ‘sea grapes’, are green seaweeds eaten raw as salad and cultivated in different parts of the world, particularly in the Indo–Pacific region [38,39]. There are several preliminary studies that have reported on the antioxidant and antibacterial effects of Malaysian green seaweeds *C. racemosa* and *C. lentillifera* [21,35,36,38,40,41], as well as the antibacterial effects of *C. racemosa* and *C. lentillifera* from India and Egypt [42,43,44,45]. The bioactive compounds responsible for the antioxidant and antibacterial effects in *C. racemosa* and *C. lentillifera*, however, are not known. This situation suggests that there is still a large gap to fill to discover the possible nature and identity of the bioactive compounds of interest from Malaysian *C. racemosa* and *C. lentillifera*.

Therefore, in the present study, we aimed to decipher the antioxidant and antibacterial properties of Malaysian *C. racemosa* and *C. lentillifera* by (1) performing phytochemical screenings to evaluate the total phenolic and flavonoid contents, (2) determining the antioxidant activity, (3) investigating the antibacterial activity, (4) partially characterizing the nature of the antibacterial effect, and (5) identifying the possible compounds present in the extract with the most promising antioxidant and antibacterial effects by liquid chromatography–mass spectrometry (LCMS) analysis. The key findings of this study will provide important insight into the antioxidant and antibacterial potentials of Malaysian green seaweeds *C. racemosa* and *C. lentillifera* to be used as novel sources of antioxidant and antibacterial compounds.

## 2. Results

### 2.1. Yield Percentage of Seaweed Extracts

Table 1 shows the amount of chloroform, methanol, and water extracts obtained from *C. racemosa* and *C. lentillifera* and their respective yield percentages. The yield percentage of the water extract of *C. lentillifera* is significantly higher (*p <* 0.05) than that of all other extracts obtained, including extracts from *C. racemosa*. On the other hand, the lowest yield percentages are from the chloroform extracts of both *C. racemosa* and *C. lentillifera*.

### 2.2. Determination of Total Phenolic Content (TPC) and Total Flavonoid Content (TFC)

The TPC in *C. racemosa* and *C. lentillifera* extracts was quantified by using the Folin Ciocalteu method. Table 2 shows a similar trend in the TPC of *C. racemosa* and *C. lentillifera* extracts, decreasing in the following order: Chloroform > methanol > water. Notably, the TPC of the chloroform extract of *C. racemosa* (13.41 ± 0.86 mg GAE/g) is significantly higher (*p <* 0.05) compared to other extracts, including those in *C. lentillifera*. In the case of *C. lentillifera*, the TPC in the chloroform extract (5.47 ± 0.75 mg GAE/g) is higher than both the water extract and the methanol extract, but it is only significantly higher (*p <* 0.05) compared to the water extract. Taken together, the TPC results suggest that both *C. racemosa* and *C. lentillifera* contain mostly of less polar phenolics.

The TFC results show that methanol extracts of both *C. racemosa* and *C. lentillifera* contain a higher amount of flavonoid compared to chloroform and water extracts. The TFC of the methanolic extract of *C. racemosa* (24.52 ± 2.17 mg QE/g) is significantly higher (*p <* 0.05) than the TFC of other extracts, including those in *C. lentillifera*. Meanwhile, in the case of *C. lentillifera*, the TFC of the methanolic extract (4.93 ± 0.27 mg QE/g) is significantly higher (*p <* 0.05) than that of the chloroform extract and the water extract by approximately 17-fold and 4-fold, respectively.

### 2.3. Determination of DPPH Radical Scavenging Activity

Results of in vitro antioxidant activity of *C. racemosa* and *C. lentillifera* are tabulated in Table 3. A lower EC_50_ value indicates a higher antioxidant activity and vice versa. In both species of seaweeds, the trend in terms of their respective antioxidant activity decreases in the following order: Chloroform > methanol > water. It is observed that the chloroform extract of *C. racemosa* shows the highest significant (*p <* 0.05) antioxidant activity (EC_50_ at 0.65 ± 0.03 mg/mL) compared with other extracts of this species. However, its antioxidant activity is significantly lower (*p <* 0.05) than that of ascorbic acid (EC_50_ at 0.01 ± 0.0005 mg/mL). Similarly, by comparing the antioxidant activity of *C. lentillifera* alone, its chloroform extract has significantly higher (*p <* 0.05) antioxidant activity (EC_50_ at 2.20 ± 0.10 mg/mL) compared to the other extracts within this species. Interestingly, by comparing across the two seaweed species, the chloroform extract of *C. racemosa* has the significantly highest (*p <* 0.05) antioxidant activity (EC_50_ at 0.65 ± 0.03 mg/mL) compared to all other extracts tested, while the water extract of *C. lentillifera* has the significantly lowest (*p <* 0.05) antioxidant activity (EC_50_ at 81.55 ± 4.22 mg/mL). 

Overall, the results of TPC of both species are in agreement with the antioxidant activity, in descending order from chloroform > methanol > water. This suggests that the antioxidant activity of the extracts may be contributed mainly by the phenolics in the extracts. The difference in trend in the TFC of *C. racemosa* and *C. lentillifera* extracts compared to the antioxidant activity and TPC indicates that the antioxidant activity in the chloroform extract may be attributed to the phenolic compounds but not to the flavonoid compounds.

### 2.4. Antibacterial Assay

#### 2.4.1. Screening of Antibacterial Effect at 250 μg/mL

Antibacterial property of the seaweed extracts was determined by performing antibacterial assay and calculating the percentage of antibacterial effect of each extract on MRSA and *E. coli* K1. Table 4 shows the percentages of antibacterial effect of each crude extract against two microorganisms.

The chloroform extract of *C. racemosa* shows the highest percentage of antibacterial effect (97.7 ± 0.30%) against MRSA compared to all other crude extracts from this seaweed species, but this extract does not show similar promising results against *E. coli* K1. The water extract from *C. racemosa* encourages the growth of both MRSA and *E. coli* K1 by −237.79 ± 18.62% and −37.00 ± 2.86%, respectively. On the other hand, chloroform extract of *C. lentillifera* gives a moderate antibacterial effect of 62.17 ± 6.60% on MRSA but poorly on *E. coli* K1 (12.42 ± 3.83%). The water extract of *C. lentillifera* encourages the growth of both microorganisms, showing a similar trend as observed in the water extract of *C. racemosa*. Also, in both species of seaweeds, the methanol extracts only show a moderate antibacterial effect against both MRSA and *E. coli* K1. Therefore, based on the trend of the antibacterial effect of the crude extracts, (1) the chloroform extract of *C. racemosa* has the most promising antibacterial effect, and (2) the antibacterial compound(s) are very effective against Gram-positive bacteria than that on Gram-negative bacteria.

#### 2.4.2. Dose-Dependent Antibacterial Effect of *C. racemosa* Chloroform Extract

**Figure 1:**
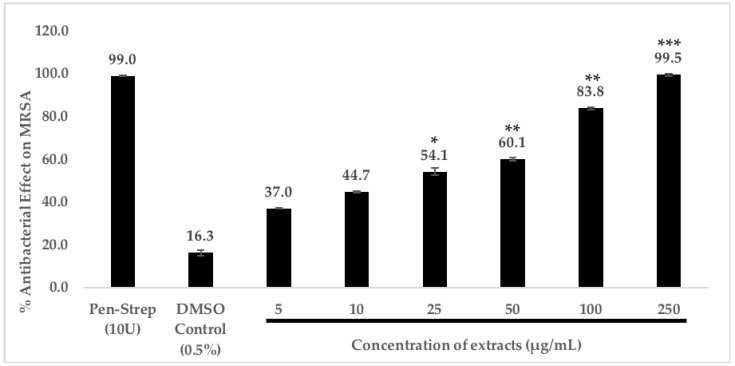
Dose-dependent antibacterial effect of *C. racemosa* chloroform extracts on MRSA. 1 × 10^6^ bacterial cells were incubated with extract at 5 μg/mL, 10 μg/mL, 25 μg/mL, 50 μg/mL, 100 μg/mL, and 250 μg/mL for 2 h at 37 °C. Next, the samples were serially diluted (10-fold) and plated on Nutrient Agar followed by 18 h of incubation at 37 °C. Finally, bacterial colonies were enumerated and recorded to obtain the % of reduction of bacterial cells. Results represent three independent experiments performed in triplicates. * indicates *p* < 0.05, ** indicates *p* < 0.01, *** indicates *p* < 0.001.

A dose-dependent antibacterial study of the chloroform extract of *C. racemosa* was performed as it showed promising antibacterial effect against MRSA. Here, we found that the antibacterial effect of *C. racemosa* chloroform extract acts in a dose-dependent manner. The highest antibacterial effect is observed at 250 μg/mL, and even at a concentration of 25 μg/mL, the extract demonstrates potent antibacterial effect against MRSA (54.1 ± 0.7%) (Figure 1). 

#### 2.4.3. Heat Treatment of *C. racemosa* Chloroform Extract

**Figure 2:**
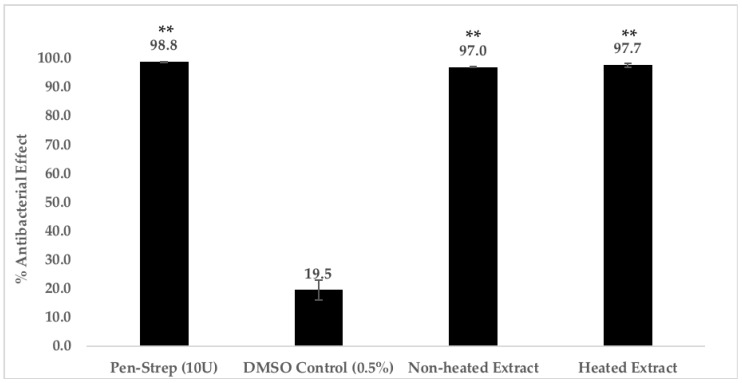
Heat treatment of *C. racemosa* chloroform extract. *C. racemosa* chloroform extract at 250 μg/mL was heated at 95 °C for 30 min prior to use for antibacterial studies. 1 × 10^6^ bacterial cells were then incubated with the heated and non-heated *C. racemosa* chloroform extract, respectively, for 2 h at 37 °C. Next, the samples were serially diluted (10-fold) and plated on Nutrient Agar followed by 18 h of incubation at 37 °C. Finally, bacterial colonies were enumerated and recorded to obtain the % of reduction of bacterial cells. Results represent three independent experiments performed in triplicates. * indicates *p* < 0.05, ** indicates *p* < 0.01, *** indicates *p* < 0.001.

In order to partially characterize the nature of the compound(s) that are responsible for the promising antibacterial effect against MRSA, we heated the *C. racemosa* chloroform extract at 95 °C for 30 min prior to use to find out if the antibacterial compound(s) are proteinaceous in nature. Based on our results, heat treatment does not alter the antibacterial effect of the chloroform extract. As shown on Figure 2, heat-treated extract exhibits 97.7 ± 0.4% of antibacterial effect, while the non-heated extract shows 97.0 ± 0.2% of antibacterial effect. The evidence indicates that the compound(s) responsible for the antibacterial effect is not proteinaceous in nature, and most likely to be small molecules.

### 2.5. Liquid Chromatography–Mass Spectrometry (LCMS) Analysis

Using LCMS and the set parameters, our LCMS results detected 74 peaks in the positive ion mass spectra, while 48 peaks were present in the negative ion mass spectra, making a total of 122 peaks, hence 122 small molecules in the *C. racemosa* chloroform extract. Twenty-three of these compounds matched with identity of known molecules on the Metlin database, with Molecular Formula Generator (MFG) scores above 90% and a ±2 difference in MFG scores. The proposed compounds were researched and are presented in Supplementary Materials Table S1. Many of these molecules are polyunsaturated and monounsaturated fatty acids, terpenes, and alkaloids. Interestingly, 25 other compounds that had MFG scores above the cut-off had no matched identity in the library, indicating a large family of new compounds that are yet to be explored in this seaweed extract (Supplementary Materials Table S2). The chromatograms and MS spectra of compounds can also be found in the Supplementary Materials Figures S1–S3.

## 3. Discussion

Seaweeds are a unique source of natural marine products due to their ability to thrive in the dynamic environment of the ocean. In the current work, we explored the antioxidant and antibacterial potentials in both *C. racemosa* and *C. lentillifera*, which are edible seaweeds widely found in the Southeast Asian countries. Although both are different species, the names ‘sea grapes’ or ‘sea caviar’ are used interchangeably by locals to describe them, and they are usually eaten raw as salad. The consumption of these sea grapes is associated with palatable taste and nutritional properties, although the latter has not been explored thoroughly [46].

The consumption of antioxidants has become an indispensable group of food products/additives due to their proven health benefits [47,48]. The antioxidant property of natural products is usually attributed by the presence of polyphenols, including phenolics and flavonoids. In this study, phytochemical tests were carried out to determine the TPC and TFC contents in crude extracts of *C. racemosa* and *C. lentillifera*. Of all the extracts, the chloroform extract of *C. racemosa* had the highest TPC value and antioxidant activity, indicating that the antioxidant activity may be associated with the phenolics in the extract. In contrast, the TFC values of *C. racemosa* had a distinct trend compared to the TPC values, suggesting that the other phenolic compounds are responsible for the antioxidant activity but not flavonoids. For instance, there are studies showing that a phenolic compound known as phlorotannin, isolated from brown seaweeds, has high antioxidant activity, which indicates that phenolic compounds do play a role in the antioxidant activity of seaweeds [49,50]. However, the identities of the phenolics responsible for the antioxidant activity from *C. racemosa* have not been reported to date, but it is an area that can be explored in the future. The positive correlation in the TPC and the antioxidant activity was also observed in the studies conducted on *C. racemosa* by Chew et al. [51], Li et al. [52], and Chia et al. [41]. As compared to other studies, the TPC of the chloroform extract of *C. racemosa* (13.41 ± 0.86 mg GAE/g) is higher than that of *Gracilaria manilaensis* (0.45 ± 0.06 mg GAE/g) [53], but lower than that of a Korean green seaweed, *Enteromorpha prolifera,* chloroform extract (80.40 ± 2.10 mg GAE/g) [54]. In comparison to several more green seaweeds from the Persian Gulf, south of Iran, the TPC of the chloroform extract of *C. racemosa* in the current study is also higher compared to the methanolic extract of *Ulva clathrata* (5.08 ± 0.65 mg GAE/g), *Ulva linza* (1.99 ± 0.29 mg GAE/g), *Ulva intestinalis* (1.98 ± 0.30 mg GAE/g), and *Ulva flexuosa* (2.67 ± 0.22 mg GAE/g) [27]. On the other hand, the DPPH radical scavenging activity obtained from the chloroform extract of *C. racemosa* in our study is higher (EC_50_ at 0.65 mg/mL) than that of a Malaysian red seaweed, *Acanthophora spicifera* (EC_50_ at 0.78 mg/mL) [55], but lower than that in another species of red seaweed, *Halopitys incurvus,* from Morocco (EC_50_ at 0.15 mg/mL) [56]. Also, when compared to another two green seaweeds, namely *Ulva expansa* and *C. sertularioides*, their methanol extracts showed weaker antioxidant activity (EC_50_ at 133.1 mg/mL and EC_50_ at 116.4 mg/mL, respectively) [57] compared to the chloroform extract of *C. racemosa* in this study. This shows that the phenolic content may vary from location to location and between different species of seaweeds, but the potential of *C. racemosa* as an antioxidant source is evident.

The second half of the present study explored the antibacterial property of *C. racemosa* and *C. lentillifera*. This is in line with the World Health Organization’s ‘Global Action Plan on Antimicrobial Resistance’ strategic objective which encourages researchers to develop new treatment options [58]. Our results suggest that these two seaweeds are potential novel sources of antibacterial compounds and these compounds are most likely heat stable. As shown earlier, the chloroform extract of *C. racemosa* has the most promising antibacterial effect against MRSA (Gram-positive bacteria) while methanol extract of *C. racemosa* has moderate antibacterial effects against *E. coli* K1 (Gram-negative bacteria). Our findings reveal that the crude seaweed extracts are more effective against Gram-positive bacteria compared to that against Gram-negative bacteria. This may be due to the difference in their cell wall structures, as well as composition [59,60]. This is similar to a study by Chan et al. [36], using the broth microdilution method, where the Gram-positive bacteria (*Bacillus cereus* and *Staphylococcus aureus*) were susceptible towards the chloroform extract of *C. racemosa* but the extract was not effective against *E. coli*. In contrast, other studies by Kandhasamy and Arunachalam [42] and Nagaraj and Osborne [44] using the disc diffusion method and the well diffusion method, respectively, reported that the methanolic extract of *C. racemosa* from India had effective antibacterial effect against both Gram-positive and Gram-negative bacteria. Also, in comparison with some Malaysian brown seaweeds from other studies using the disc diffusion method, Gram-positive bacteria (*Bacillus subtilis* and *S. aureus*) and Gram-negative bacteria (*Pseudomonas aeruginosa* and *E. coli*) were not susceptible towards chloroform extracts of *Sargassum plagyophillum*, *Sargassum flavellum*, *Padina australis,* and *Sargassum binderi* [34]. However, the Gram-positive bacteria were mostly susceptible towards the methanolic, acetone, and ethyl acetate extracts of *Sargassum plagyophillum*, *Sargassum flavellum*, and *Sargassum binderi* [34]. Interestingly, a study conducted in Saudi Arabia using the disc diffusion method showed that the chloroform extract of another member of the *Caulerpa* genus, *Caulerpa occidentalis,* was very effective against *E. coli* but only moderately against *S. aureus* and *Enterococcus faecalis* [61]. The difference in our results with other studies suggests that apart from the seaweed species tested, there are other factors that may influence the synthesis of desired bioactive compounds such as geographical localization of the seaweeds and method of extraction [42].

Our data suggest that the chloroform extract of *C. racemosa* is a potent antioxidant and antibacterial agent, and the antibacterial effect might be attributed to a bioactive compound(s) that is non-heat sensitive. LCMS data reveal a wide spectrum of chemical classes, including polyunsaturated and monounsaturated fatty acids, terpenes, and alkaloids, that are potentially present in the extract (Supplementary Materials Table S1). Out of the 23 proposed compounds (with MFG scores above 90%), only Pristimerin (Compound 1) has been reported to have antioxidant activity [62], while Pheophorbide a (Compound 12) has been reported with antioxidant and antibacterial effects [63,64], which could be responsible for the observed potent antibacterial effect, as well as antioxidant activity. However, there are 25 more compounds (Supplementary Materials Table S2) from the extract that have no matched identity on the library, which may be novel compounds that are medically essential, but further characterization is needed to determine their uses. According to Yang et al. [3], Guerriero et al. [65], and Smyrniotopoulos et al. [66], *Caulerpa* genus seaweeds synthesize a plethora of metabolites from the bisindole alkaloids and terpenoids classes (including sesquiterpenoids and diterpenoids), which support our findings, where compounds such as Compound 2 (Caulerpin, a bisindole alkaloid), Compound 4 (Isoamijiol, a diterpenoid), and Compound 23 (Methyl farnesoate, a sesquiterpenoid) are identified from the chloroform extract of *C. racemosa*. In fact, Caulerpin was successfully isolated from *C. racemosa* and *C. lentillifera* (harvested in Sabah, Malaysia) and identified as the compound responsible for the potent antibacterial effect in green seaweeds [38]. Therefore, it is believed that Caulerpin might play a role in the antibacterial effect in *C. racemosa* and *C. lentillifera,* but it is still not clear if this is the only compound exhibiting the antibacterial effect. Interestingly, the compounds listed in Supplementary Materials Table S1 are not phenolics but are mostly polyunsaturated fatty acids (PUFA) such as Compound 6 (12(13)-epoxy-9Z,15Z-octadecadienoic acid), Compound 7 (9Z,12Z,15E-octadecatrienoic acid), and Compound 9 (trans-5, trans-8, trans-11-hexadecatrienoic acid), to name a few. As far as we are concerned, the bioactivity of these PUFAs has not been reported yet, but there are studies showing that PUFAs have antioxidant property [67,68] and antibacterial property [69,70,71,72]. However, it is also possible that there are phenolics in various amounts among the unidentified 25 compounds found in the extract and may be collectively contributing to the antioxidant effect observed here. Overall, only a little is known about the antioxidant and antibacterial properties of the compounds listed in Supplementary Materials Table S1. Our preliminary LCMS analysis suggests that further investigation is required. In the near future, tandem LCMS (LCMS/MS) can be used to reveal the small molecules present in the chloroform extract of *C. racemosa* with a higher level of confidence.

Seaweeds are found under the sea, a challenging environment with constant exposure to various microorganisms [73]. The search for novel antibacterial compounds in marine seaweeds has been of interest to various research groups around the world. Studies on the antibacterial effect of red and brown seaweeds have already attracted much attention from scientists, but such studies in green seaweeds are still lacking [1]. For instance, eight novel potent antibacterial compounds against MRSA identified as Chrysophaentins A–H, were successfully isolated from a golden seaweed, *Chrysophaeum taylori* [74], while Bromophycolides P and Q with strong antibacterial effect against vancomycin-resistant *Enterococcus faecium* (VREF) were isolated form a red seaweed, *Callophycus serratus* [75]. These findings have demonstrated that seaweeds are a potential source of novel antibacterial compounds and should be investigated in the discovery of new therapeutic drugs against ARB.

The current study examined the susceptibility of two pathogenic bacteria, MRSA and *E. coli* K1, representing Gram-positive and Gram-negative bacteria, respectively, towards the seaweed extracts. MRSA is an ARB that can cause severe skin infections and if left untreated, may lead to sepsis. In 2005 alone, more than 18,000 deaths were reported in the U.S. [76] and the number of MRSA-associated hospitalization cases doubled from 1998 to 2007 in the U.S., suggesting that the infection has become more prevalent over the years [77]. Compounding the issue, MRSA has been reported to acquire resistance against newer antibiotics intended for MRSA such as daptomycin-, vancomycin-, and teicoplanin [78,79,80]. The selected Gram-negative bacterium, *E. coli* K1, is a prevalent causative agent for neonatal meningitis. The emergence of extended-spectrum beta lactamase (ESBL) [81] and carbapenem-resistant *E. coli* strains [82] are on the rise, further reducing the therapeutic options for patients. The findings of the present study undoubtedly shed light on the development of new antibacterial agents against these deadly pathogens.

The extracts from each of the seaweeds exerted different strengths of antioxidant and antibacterial effects, possibly due to the difference in the bioactive compounds present in each extract, growth stage of the seaweeds, and seasonal variations [1]. By referring to the yield percentages of the extracts, the water extract of *C. lentillifera* has the highest yield. This demonstrates that *C. lentillifera* possesses a high content of more polar compounds, which are effectively extracted by water. While a variety of solvents have been used to prepare seaweed extracts for antioxidant and antibacterial testing, there are still no optimal solvents to prepare an extract with the most effective antioxidant or antibacterial activity in seaweeds, because numerous factors such as the species of seaweed used and the species of bacteria we are targeting need to be taken into consideration [1]. For instance, Cox et al. [83] suggest that methanol is a better solvent to prepare extract with promising antibacterial property in brown seaweed, while acetone is effective for red and green seaweeds. In contrast, Moubayed et al. [84] recommend methanol to extract effective antibacterial compounds from brown and green seaweeds. As for the extraction of effective antioxidant compounds, Airanthi et al. [85] found that methanol is the most effective solvent in extracting antioxidant compounds from brown seaweeds, while Chakraborty and Raola [86] found that using ethyl acetate for the extraction of antioxidant compounds from red seaweeds is more effective compared to dichloromethane and hexane. Nevertheless, a suitable solvent for the most effective extraction of antioxidant and antibacterial compounds is still vague, and further studies in this area are needed to increase our understanding on the choice of solvent for effective extraction.

Intriguingly, the water extract of both seaweeds encouraged the growth of both the bacteria tested, showing a possibility that water extracts contain nutrients that can support the growth of the bacteria. For example, according to Egan et al. [87], the cell wall or other carbon-rich polymers (e.g., cellulose and alginate) in seaweed may serve as a source of nutrients for bacteria that are capable of utilizing them, thus supporting our hypothesis. These nutrients may be polar in nature and can be extracted by water. Similar to another study by Kantachumpoo and Chirapart [4], the water extracts from seven species of brown seaweed were not only inactive against all of the Gram-positive and Gram-negative bacteria tested, but instead promoted their growth. This trend suggests that although seaweeds have potent antibacterial property, they also have compounds that are able to support the growth of bacteria regardless of their species. In recent years, the prebiotic potential of seaweeds has been explored [2]. The water extracts of *C. racemosa* and *C. lentillifera* may be evaluated for their prebiotic potentials due to their potential to promote the growth of bacteria tested here.

Several environmental factors such as the temperature and salinity of the seawater fluctuate from time to time which, in turn, affects the biology of the seaweeds. Ding et al. [88] reported that an increase in water temperature from 15 °C to 25 °C led to an increase in the growth rates of a red seaweed known as *Hypnea cervicornis.* However, the increased in temperature to 30 °C resulted in wilted seaweed. In the case of salinity, the increase of NaCl concentration from 25% to 45% significantly increased the synthesis of a light-harvesting pigment known as Car pigment at 20 °C. Therefore, we cannot exclude the possibility that the antioxidant and antibacterial effects in the seaweeds will not fluctuate with the change in certain environmental factors. Further investigation focusing on the antioxidant and antibacterial effects throughout the year can be explored to determine the fluctuations in those properties. Besides, the promising antibacterial effects of *C. racemosa* is encouraging to us to proceed by exploring the cytotoxicity of the extract, because it is crucial for a compound to be non-cytotoxic and have promising desired effects at the same time. Although we observed promising antibacterial effect from the extract, the mechanism of action of the antibacterial effect is still not known. Therefore, future works should focus on isolating and purifying the compound(s) responsible for the desired effects, as well as the mechanism of action. In addition, our study only utilized one Gram-positive and one Gram-negative bacteria to represent the two groups of bacteria in their susceptibility towards the seaweed extracts. This may underrepresent the results and the potential of the seaweeds as a source of potent antibacterial compound(s). In order to determine the antibacterial effects of the extracts at a broader spectrum, more species of bacteria should be included in the antibacterial studies.

In addition, future works should also explore the possibility of bacteria associated with *C. racemosa* and *C. lentillifera* to investigate if they are responsible for the synthesis of antibacterial compounds. This is because there are studies that have reported that seaweed-associated bacteria play crucial roles in the development and growth of seaweeds, showing a symbiotic relationship between the bacteria and the seaweeds [89]. Another study found a potential broad-spectrum antibacterial effect from epiphytic bacteria (Phylum: Firmicutes; closely related to *Bacillus pumilus*) isolated from brown seaweed *Padina pavonica,* thus suggesting that bacteria associated with seaweeds might be producers of antibacterial compounds [90]. Recently, a study by Sujuliyani et al. [91] showed that *C. racemosa* has symbiotic bacteria belonging to the genus *Neisseria* that is able to synthesize potential antibacterial compounds against *S. aureus* and *Salmonella typhi*. Therefore, it is possible that symbiotic bacteria of seaweeds produce compounds with antibacterial effect, protecting the seaweeds from other harmful microorganisms.

## 4. Materials and Methods 

### 4.1. Seaweed Preparation

Fresh specimens of *C. racemosa* and *C. lentillifera* were collected from Port Dickson coastal area (GPS Coordinate: N 2.413029; E 101.855892) in July 2017. The specimens were washed with sea salt water and gently brushed to remove foreign objects and epiphytes. The seaweeds were then rinsed with distilled water to remove excessive salts before storing at −20 °C. Next, seaweeds were freeze-dried (Labogene SCANVAC Coolsafe Touch 110-4, Denmark) and then ground into fine powder with a food grinder and kept at –20 °C prior to use.

### 4.2. Crude Extract Preparations

Seaweed extraction was performed via simple extraction with three solvents: Chloroform, methanol, and water. Briefly, 1 g of seaweed powder of each species was incubated with the appropriate solvent at room temperature for 2 h. The filtrates obtained were subjected to rotary evaporation (Fisher Scientific EYELA N-1200A Rotary Evaporator, Tokyo) and further concentrated using vacuum concentrator (SpeedScan 40, Korea) at 200 rpm. Finally, the dried extracts were stored at −20 °C. Crude extracts were prepared in three separate independent extractions. The yield percentage was calculated based on the dried mass of the extract and the initial weight of the powder used for extraction.

### 4.3. Phytochemical Screening

#### 4.3.1. Determination of Total Phenolic Content (TPC)

The total phenolic content (TPC) of chloroform, methanol, and water extracts of *C. racemosa* and *C. lentillifera* was measured by using the Folin–Ciocalteu method, as described by Singleton et al. [92], with slight modifications. Briefly, 5 µL of 10 mg/mL from of each extract and gallic acid (as standard) was mixed with 25 µL of FC reagent. Next, 395 µL of distilled water was added followed by 75 µL of 20% Na_2_CO_3_. The contents in the samples and standards were mixed thoroughly and allowed to stand in dark for 60 min. Absorbance was read at 750 nm by using a spectrophotometer microplate reader (Infinite 200 Pro, Tecan, Switzerland). The TPC values were calculated by the graphical method and are expressed as milligram gallic acid equivalent per gram (mg GAE/g). All tests and controls were tested in triplicates in three separate independent experiments.

#### 4.3.2. Determination of Total Flavonoid Content (TFC)

The total flavonoid content (TFC) of chloroform, methanol, and water extracts of *C. racemosa* and *C. lentillifera* was measured according to the method described by Pękal and Pyrzynska [93], with slight modifications. Briefly, 10 µL of 10 mg/mL from of each extract and Quercetin (as standard) (Sigma Life Sciences, Germany) was mixed with 250 µL of 2% aluminum chloride (Sigma-Aldrich, Germany). Next, 250 µL of 1 M acetic acid was added followed by 490 µl of distilled water. The contents in the samples and standards were mixed thoroughly and allowed to stand in dark for 15 min. Absorbance was read at 425 nm by using a spectrophotometer microplate reader (Infinite 200 Pro, Tecan, Switzerland). The TFC values were calculated by the graphical method and are expressed as milligram quercetin equivalent per gram (mg QE/g). All tests and controls were tested in triplicates in three separate independent experiments.

### 4.4. Determination of DPPH Radical Scavenging Activity

The antioxidant activity of extracts was determined using the 2,2-diphenyl-1-picrylhydrazyl (DPPH) scavenging activity as described by Pang et al. [94], with slight modifications. A positive control was prepared using Ascorbic acid (Sigma, Germany). Briefly, 50 µL of the seaweed samples and the positive control was mixed with 1 mL of 0.1 mM DPPH (Alfa Aesar, U.S), followed by incubation in the dark for 30 min. The absorbance was then measured using spectrophotometer (Tecan, Switzerland) at 518 nm. All tests and controls were tested in three separate independent experiments. The DPPH radical scavenging activity percentage was calculated with Equation (1) stated below:

(1) $$\mathrm{DPPH}\;\mathrm{radical}\;\mathrm{scavenging}\;\mathrm{activity}\;\left(\%\right)=\left(1-\frac{\mathrm{Absorbance}\;\mathrm{of}\;\mathrm{samples}\;\mathrm{at}\;518\mathrm{nm}}{\mathrm{Absorbance}\;\mathrm{of}\;\mathrm{negative}\;\mathrm{control}\;\mathrm{at}\;518\mathrm{nm}}\right)\times 100\%$$

The antioxidant results are expressed as half-maximum effective concentration (EC_50_), calculated by utilizing the dose-dependent curve of percentage of DPPH radical scavenging activity versus the concentrations of the respective samples/standards.

### 4.5. Antibacterial Assay

#### 4.5.1. Screening of Antibacterial Effect at 250 μg/mL

Antibacterial assays were carried out as previously described by Lee et al. [95] and Khan et al. [96] with slight modifications to determine the antibacterial effect of the seaweed extracts. Gram-positive bacteria Methicillin-resistant *Staphylococcus aureus* (MRSA) (MTCC 381123) and Gram-negative bacteria *Escherichia coli* K1 (MTCC 710859) were used in this study. The test bacteria were grown in Nutrient Broth for 18 h at 37 °C prior to use. Approximately 1 × 10^6^ CFU (10 μL) of cells were incubated with 1 μL of seaweed extract at a final concentration of 250 μg/mL in 189 μL of PBS followed by incubation for 2 h at 37 °C. The extracts were diluted in their respective solvent, except for chloroform extract, which was dissolved in dimethyl sulfoxide (DMSO). The incubated cultures were then serially diluted (10-fold) in sterile distilled water and plated on nutrient agar plates. The plates were incubated overnight at 37 °C and the bacterial colonies were then manually enumerated and recorded. Penicillin-streptomycin (10 U) was used as the positive control, whereas solvent controls were prepared by incubating the bacterial cultures in 0.5% of the appropriate extract solvent (methanol, water, or DMSO). All tests and controls were tested in triplicates in three separate independent experiments. The formula used to calculate the CFU/mL of the bacteria is stated as Equation (2) while the percentage of antibacterial effect based on the CFU/mL was calculated using the Equation (3) stated below:

(2) $$\mathrm{CFU}/\mathrm{mL}=\frac{\mathrm{No.}\;\mathrm{of}\;\mathrm{colonies}\times \mathrm{Dilution}\;\mathrm{factor}}{0.01\;\mathrm{mL}}$$

(3) $$\mathrm{Percentage}\;\mathrm{of}\;\mathrm{antibacterial}\;\mathrm{effects}\;\left(\%\right)=\frac{\frac{\mathrm{CFU}}{\mathrm{mL}}\;\mathrm{of}\;\mathrm{Nontreated}\;\mathrm{sample}-\frac{\mathrm{CFU}}{\mathrm{mL}}\;\mathrm{of}\;\mathrm{Treated}\;\mathrm{sample}}{\frac{\mathrm{CFU}}{\mathrm{mL}}\;\mathrm{of}\;\mathrm{Nontreated}\;\mathrm{sample}}\times 100\%$$

#### 4.5.2. Dose-Dependent Antibacterial Effect of *C. racemosa* Chloroform Extract

The antibacterial assays were conducted as mentioned earlier but with different concentrations of chloroform extracts of *C. racemosa* to investigate if the antibacterial effect exhibited by chloroform extract of *C. racemosa* acts in a dose-dependent manner. The final concentrations of the extract tested were 5, 10, 25, 50, 100, and 250 μg/mL. All tests and controls were tested in triplicates in three separate independent experiments.

#### 4.5.3. Heat Treatment of *C. racemosa* Chloroform Extract

Heat treatment on the chloroform extract of *C. racemosa* was conducted to determine if the bioactive compound(s) responsible for the antibacterial effect is proteinaceous in nature. Briefly, the antibacterial assay was performed as elucidated earlier but the extract was heated at 95 °C for 30 min prior to use for antibacterial studies. All tests and controls were tested in triplicates in three separate independent experiments.

### 4.6. Liquid Chromatography–Mass Spectrometry (LCMS) Analysis

#### 4.6.1. Liquid Chromatography–Mass Spectrometry (LCMS): Separation and Analysis

The *C. racemosa* chloroform extract was analyzed using a LCMS on Agilent 1290 infinity liquid chromatograph (Agilent Technologies, Wilminton, DE, USA), coupled with an Agilent 6520 Accurate-Mass Q-ToF Mass Spectrometer with dual ESI source. Separation of compound was achieved using reverse-phase HPLC with an Agilent Zorbax Eclipse XDB-C18 column, Narrow-Bore 2.1 × 150 mm, of particle size 3.5 µm at 25 °C, and equilibrated with solvent A (0.1% formic acid in Milli-Q water) and solvent B (0.1% formic acid in acetonitrile). The flow rate was set at 0.5 mL per min. The total run time was 30 min, including a 25 min run time, and a 5 min post-run time. The ESI-TOF/MS conditions were optimized as follows: Drying gas temperature, 300 °C; drying gas flow, 10 L/min; nebulizer gas pressure, 45 psi; capillary voltage, 3500 V for positive ion mass spectra and 4000 V for negative ion mass spectra; fragmentation voltage, 125 V; and skimmer, 65 V. The mass spectrum was scanned from m/z 100 to m/z 3200 in both the positive and negative ionization modes. Calibration reference solutions obtained from Agilent were used to calibrate the mass spectrometer daily. Reference solution was used and the two ions with m/z of 119.03632 and 966.0097 were selected for mass calibration in order to eliminate systematic errors.

#### 4.6.2. Identification of Compounds Through Matching with Library

Chloroform extract of *C. racemosa* was subjected to LCMS to obtain the chromatograms and the prospective mass spectra of every separated fraction of the mixture of compounds. The MS spectra for the compounds present in the extract were run against the NIST Mass Spectral Search Program-2009 version 2.0f (National Institutes of Standard and Technology, Gaithersburg, MD, USA) for the identification of homologous compounds through Agilent MassHunter Qualitative Analysis B.05.00 software.

### 4.7. Statistical Analysis

All data collected were in triplicates of three independent experiments, except yield percentage, DPPH radical scavenging activity, and LCMS analysis. The results are presented as mean ± standard error (SE). The statistical analysis of antibacterial studies was carried out using student’s *t*-test (two-tailed). The results of the yield percentage, phytochemical assays, and DPPH radical scavenging activity were subjected to one-way analysis of variance (ANOVA) with post-hoc tests (Tukey for yield percentage and TPC; Games–Howell for TFC and DPPH radical scavenging activity) using SPSS Statistics Version 23 for the analysis of significant differences (*p* < 0.05) in the properties tested between extracts.

## 5. Conclusions

In conclusion, *C. racemosa* is shown to have promising antioxidant potential and antibacterial effect against Gram-positive bacteria. Our findings also reveal that heat-treatment on the chloroform extract of *C. racemosa* does not alter the antibacterial effect, suggesting that the antibacterial compound(s) in the extract is not proteinaceous in nature but could be non-thermolabile small metabolites. Besides, we also observed a positive correlation between the TPC, antioxidant, and antibacterial activities from *C. racemosa* suggesting that these two properties may be contributed by phenolics but not flavonoids, due to the difference in trend in the TFC results. Nevertheless, *C. racemosa* may be a novel source for antioxidant and antibacterial compounds and has high potential to be harnessed for the development of pharmaceutically useful drugs. Further and more in-depth studies are required to build our understanding on the mechanism of action, identity of the specific compound(s) responsible for the desired effects, and also to expand our knowledge on the other hidden potentials in *C. racemosa*.

## 6. Patents

No patents resulting from the work reported in this manuscript.

## Figures and Tables

**Table 1:** Yield percentages of seaweed extracts from each seaweed species.

Seaweed Species	Extract	Mass of Extract Obtained (g)	Yield (%)
	Chloroform	0.053 ± 0.001	5.212 ± 0.106 ^a^
*C. racemosa*	Methanol	0.126 ± 0.004	12.554 ± 0.434 ^b^
	Water	0.133 ± 0.002	12.920 ± 0.289 ^b^
	Chloroform	0.052 ± 0.005	5.081 ± 0.462 ^a^
*C. lentillifera*	Methanol	0.138 ± 0.015	13.450 ± 1.429 ^b^
	Water	0.319 ± 0.020	30.884 ± 2.006 ^c^

Data show the mass of seaweed extracts obtained and their respective yields. Extracts were prepared by incubating 1 g of seaweed powder with the appropriate solvent (50 mL) for 2 h. The filtrates collected were subjected to rotary evaporation and then further concentrated using vacuum concentrator. The extracts obtained were stored at −20 °C prior to use. Data are expressed as mean ± SE of three separate independent extractions. Means with different alphabet are significantly different at *p* < 0.05 (Tukey).

**Table 2:** The total phenolic content (TPC) and total flavonoid content (TFC) in *C. racemosa* and *C. lentillifera* crude extracts.

Seaweed Species	Extract	TPC (mg GAE/g)	TFC (mg QE/g)
	Chloroform	13.41 ± 0.86 ^a^	5.46 ± 0.41 ^a,c,e^
*C. racemosa*	Methanol	10.33 ± 0.02 ^b^	24.52 ± 2.17 ^b^
	Water	1.74 ± 0.09 ^c^	2.50 ± 0.10 ^a,c^
	Chloroform	5.47 ± 0.75 ^d^	0.28 ± 0.03 ^d^
*C. lentillifera*	Methanol	4.52 ± 0.42 ^d^	4.93 ± 0.27 ^a,e^
	Water	2.04 ± 0.36 ^c^	1.17 ± 0.03 ^f^

Data are expressed as mean ± SE of triplicates in three separate independent experiments. TPC values are expressed as milligram gallic acid equivalent per gram (mg GAE/g). TFC values are expressed as milligram Quercetin equivalent per gram (mg QE/g). Means with different alphabet are significantly different at *p* < 0.05 (Tukey for TPC; Games–Howell for TFC).

**Table 3:** The DPPH radical scavenging activity of *C. racemosa* and *C. lentillifera* crude extracts.

Standard and Seaweed Species	Control and Extract	EC_50_ (mg/mL)
Standard	Ascorbic Acid	0.01 ± 0.0005 ^a^
	Chloroform	0.65 ± 0.03 ^d^
*C. racemosa*	Methanol	2.51 ± 0.09 ^c,g^
	Water	7.46 ± 0.20 ^b,f^
	Chloroform	2.20 ± 0.10 ^c,g^
*C. lentillifera*	Methanol	9.74 ± 0.59 ^b,f^
	Water	81.55 ± 4.22 ^e^

Data are expressed as mean ± SE of three separate independent experiments. Means with different alphabet are significantly different at *p* < 0.05 (Games–Howell).

**Table 4:** Percentage of antibacterial effect of seaweed crude extracts against MRSA and *E. coli* K1.

Seaweed Extracts and Controls	Antibacterial Effect (%)	
	MRSA	*E. coli* K1
***C. racemosa* Extract**		
Chloroform	97.70 ± 0.30 *	19.90 ± 4.05
Methanol	61.54 ± 2.19 ***	42.91 ± 7.75
Water	−237.79 ± 18.62 ***	−37.00 ± 2.86 **
***C. lentillifera* Extract**		
Chloroform	62.17 ± 6.60 *	12.42 ± 3.83
Methanol	44.99 ± 2.42 **	42.26 ± 12.67 *
Water	−34.58 ± 2.71	−43.05 ± 0.99 ***
**Controls**		
Pen-strep (10 U)	98.10 ± 0.10	100.00 ± 0.0
DMSO (0.5%)	15.90 ± 1.70	10.90 ± 3.70
Methanol (0.5%)	8.70 ± 1.56	11.80 ± 3.00
Water (0.5%)	−28.30 ± 5.68	−9.72 ± 0.92

Percentage of antibacterial effect of seaweed crude extracts against MRSA and *E. coli* K1. 1 × 10^6^ bacterial cells (10 μL) were incubated with extract at 250 μg/mL (1 μL) in 189 μL of phosphate-buffered saline (PBS) for 2 h at 37 °C. Next, the samples were serially diluted (10-fold) and plated on Nutrient Agar followed by 18 h of incubation at 37 °C. Finally, bacterial colonies were enumerated and recorded to obtain the percentage of reduction of bacterial cells. The values represent mean ± SE of triplicate in three separate independent experiments. *p*-values of * *p* < 0.05, ** *p* < 0.01, and *** *p* < 0.001 were calculated using student’s *t*-test by comparing with the respective solvent control. Abbreviations: DMSO, dimethyl sulfoxide; Pen-strep, penicillin-streptomycin.

## Supplementary Materials

The following are available online at https://www.mdpi.com/2079-6382/8/3/152/s1: Table S1. Proposed compounds present in *C. racemosa* chloroform extract identified via LCMS analysis; Table S2. Molecular formula of 25 unknown compounds from *C. racemosa* chloroform extract; Figure S1. *C. racemosa* chloroform extract was subjected to LCMS qualitative analysis using negative ion mode; Figure S2. *C. racemosa* chloroform extract was subjected to LCMS qualitative analysis using positive ion mode; Figure S3. Mass spectra of individual compounds listed in Supplementary Material Table S1.
