# Supplementary material for: Decoding Antioxidant and Antibacterial Potentials of Malaysian Green Seaweeds: Caulerpa racemosa and Caulerpa lentillifera
Source: Antibiotics (Basel). 2019 Sep 17;8(3):152. doi: 10.3390/antibiotics8030152 (PMC6783820; doi:10.3390/antibiotics8030152)
Supplement: Supplementary file 1 [file antibiotics-08-00152-s001.pdf]

Supplementary Material Table S1: Proposed compounds present in *C. racemosa* chloroform extract identified via LCMS analysis.

No.	Compound name	Formula	Structure	Reported activity
1	Pristimerin; Celastrol methyl ester	$C_{30}H_{40}O_4$	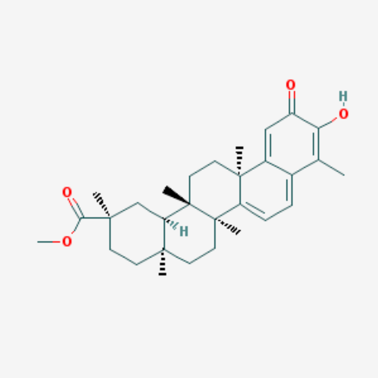	Pristimerin is a triterpenoid commonly found in various members from the Celestraceae family, including *Maytenus ilicifolia*. Anticancer activities of Pristimerin have been reported in uveal melanoma [1], glioma [2], colorectal cancer [3], osteosarcoma [4], cervical cancer [5], ovarian cancer [62], pancreatic cancer [6, 7], prostate cancer [8, 9], and breast cancer [10, 11].<br><br>Pristimerin has also been shown to have anti-inflammation effects [12-14] and antioxidant activities [62]. Very little is known about its antibacterial effects but this triterpenoid has been shown to inhibit SARS-CoV and cytomegalovirus *in vitro* [15, 16].
2	Caulerpin	$C_{40}H_{18}N_2O_4$	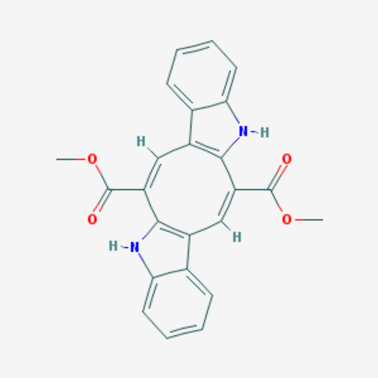	Caulerpin is a red pigmented bis-indole alkaloid isolated from marine *Caulerpa* algae spp. This molecule has been demonstrated to have anticancer effects [17-19]. It is also known to have antinociceptive effects [20, 21]. This alkaloid is known to possesses antibacterial activity [44] and anti-tuberculosis activity [22].

3	5(S)-HETE lactone	$C_{20}H_{30}O_2$	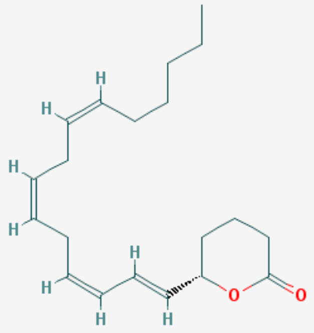	Bioactivity has not been reported.
4	Isoamijiol	$C_{20}H_{30}O_2$	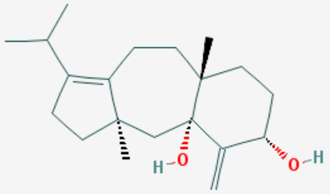	Isoamijiol is a diterpenoid. This molecule is commonly found in brown algae of genus *Dictyota* [23]. Its bioactivity is not known.
5	12-oxo-10Z-octadecenoic acid	$C_{18}H_{32}O_3$	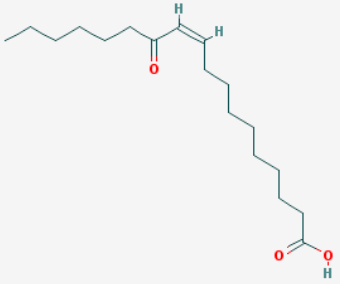	Bioactivity has not been reported.

6	12(13)-epoxy-9Z,15Z-octadecadienoic acid or α-9(10)-EpODE	$C_{18}H_{30}O_3$	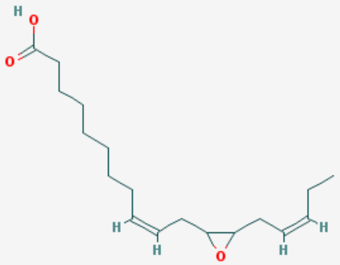	Bioactivity has not been reported.
7	9Z,12Z,15E-octadecatrienoic acid	$C_{18}H_{30}O_2$	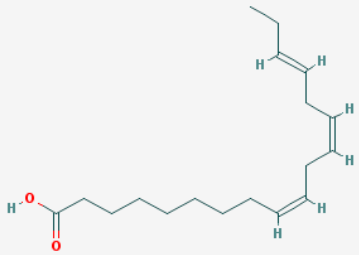	Bioactivity has not been reported.
8	4E,10Z-Tetradecadienyl acetate	$C_{16}H_{28}O_2$	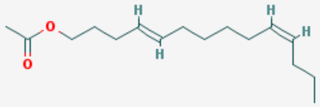	A sex pheromone found in Apple Leafminer Moth, *Phyllonorycter ringoniella* [24].

9	trans-5, trans-8, trans-11-hexadecatrienoic acid	$C_{16}H_{26}O_2$	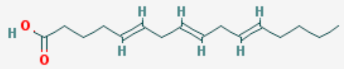	Bioactivity has not been reported.
10	7-Octadecenoic acid	$C_{18}H_{32}O_2$	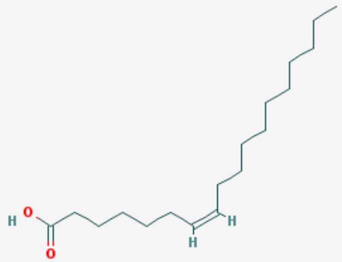	This monounsaturated fatty acid is found in pistachio nuts [25] but its bioactivity is not known.
11	Harderoporphyrin	$C_{35}H_{36}N_4O_6$	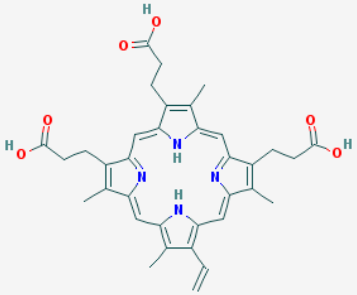	Harderoporphyrin was first identified and isolated from rat Harderian glands. Its presence and amount in the rodent harderian glands has been studied to be used as a biomarker for arsenic exposure [26].

12	Pheophorbide a	$C_{35}H_{36}N_4O_5$	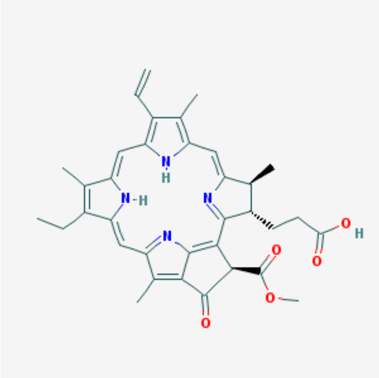	This bioactive molecule has been isolated from marine algae species and terrestrial plants. It is known to have antioxidant and anti-inflammation effects [63].<br><br>This molecule also induces photodynamic inactivation of *Trypanosoma cruzi* [27]. It is also known to have anti-*S. aureus* [64] and anti-hepatitis C virus [28].<br><br>On its own, pheophorbide-a has been shown to induce apoptosis in human hepatocellular carcinoma cells [29].
13	Micromonal	$C_{40}H_{56}O_3$	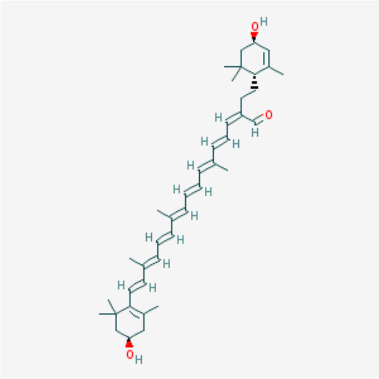	This carotenoid is found in green algae Prasinophyceae [30].

14	4-Ketolutein D and 4-ketolutein F	$C_{40}H_{54}O_3$	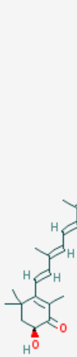 4-ketolutein-D 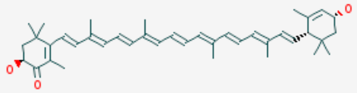 4-ketolutein-F	4-ketolutein is a ketolated carotenoid found in algae and red tilefish (*Branchiostegus japonicas*) [31]. This carotenoid is now patented to as a food additive in egg yolk [32].
15	Diatoxanthin/ 7,8-Didehydrozeaxanthin	$C_{40}H_{54}O_2$	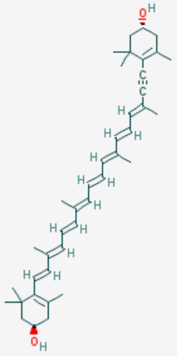	Diatoxanthin is a carotenoid in algae that plays a role in dissipating excess energy in algae species [33]. This carotenoid has been shown to have anti-inflammation in the LPS-induced pro-inflammatory in RAW264.7 cells [34].

16	Pectenoxanthin; Alloxanthin; Manixanthin;	$C_{40}H_{52}O_2$	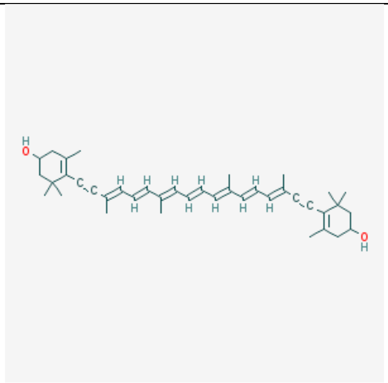	Alloxanthin is a carotenoid that has been isolated from various marine species, including sea pineapple (*Halocynthia roretzi*), channel catfish, giant scallop (*Pecten maximus*) and edible mussel (*Mytilus edulis*) [35]. Similarly, to Diatoxanthin, this carotenoid was shown to be anti-inflammation *in vitro* [34].
17	1α-hydroxy-24-methylvitamin D2 / 1α-hydroxy-24-methylergocalciferol	$C_{29}H_{46}O_2$	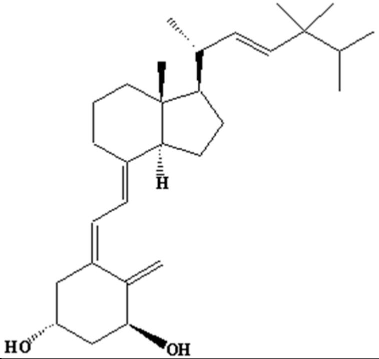	Vitamin D.
18	Coprocholic acid; 3α,12α-Dihydroxy-5β-chol-8(14)-en-24-oic Acid	$C_{24}H_{38}O_4$	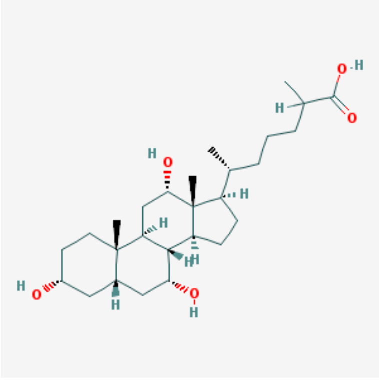	Coprocholic acid, also called 3α, 7α, 12α-Trihydroxy-5β-cholestan-26-oic acid, is a bile acid.

19	5Z,8Z,11Z,14Z-Octadecatetraenoic acid	$C_{18}H_{30}O_2$	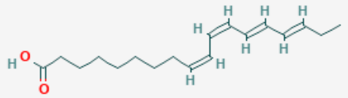	Bioactivity has not been reported.
20	9Z,12Z,15E-octadecatrienoic acid	$C_{18}H_{30}O_2$	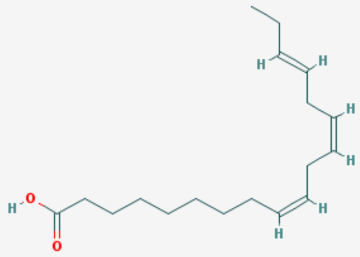	Bioactivity has not been reported.
21	5Z,8Z,11Z,14Z-octadecatetraenoic acid	$C_{18}H_{28}O_2$	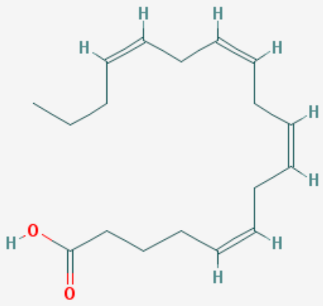	Bioactivity has not been reported.

22	10E, 12Z-Tetradecadienyl acetate	$C_{16}H_{28}O_2$	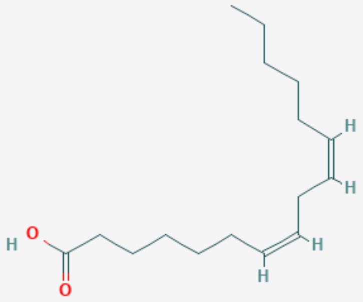	A sex pheromone found in the avocado pest, *Amorbia cuneana* (Walsingham) [36].
23	Methyl farnesoate	$C_{16}H_{26}O_2$	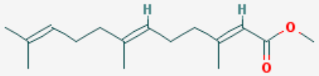	A sesquiterpenoid hormone that is found in the *Drosophila* metamorphosis and stingless bee [37, 38].

Reference List for Supplementary Material Table S1: Proposed compounds present in *C. racemosa* chloroform extract identified via LCMS analysis.

1. Zhang, B.; Zhang, J.; Pan, J. Pristimerin effectively inhibits the malignant phenotypes of uveal melanoma cells by targeting NF-κB pathway. *Intl J Oncol* **2017**, *51*, 887 - 898. DOI: 10.3892/ijo.2017.4079.
2. Zhao, H.; Wang, C.; Lu, B.; Zhou, Z.; Jin, Y.; Wang, Z.; Zheng, L.; Liu, K.; Lou, T.; Zhu, D.; et al. Pristimerin triggers AIF-dependent programmed necrosis in glioma cells via activation of JNK. *Cancer Lett* **2016**, *374*, 136 - 148. DOI: 10.1016/j.canlet.2016.01.055.
3. Yousef, B.A.; Hassan, H.M.; Guerram, M.; Hamdi, A.M.; Wang, B.; Zhang, L.Y.; Jiang, Z.Z. Pristimerin inhibits proliferation, migration and invasion, and induces apoptosis in HCT-116 colorectal cancer cells. *Biomed Pharmacother* **2016**, *79*, 112 - 119. DOI: 10.1016/j.biopha.2016.02.003.
4. Mori, Y.; Shirai, T.; Terauchi, R.; Tsuchida, S.; Mizoshiri, N.; Hayashi, D.; Arai, Y.; Kishida, T.; Mazda, O.; Kubo, T. Antitumor effects of pristimerin on human osteosarcoma cells *in vitro* and *in vivo. Onco Targets Ther* **2017**, *10*, 5703 – 5710. DOI: 10.2147/OTT.S150071.
5. Eum, D.Y.; Byun, J.Y.; Yoon, C.H.; Seo, W.D.; Park, K.H.; Lee, J.H.; Chung, H.Y.; An, S.; Suh, Y.; Kim, M.J.; Lee. S.J. Triterpenoid pristimerin synergizes with taxol to induce cervical cancer cell death through reactive oxygen species-mediated mitochondrial dysfunction. *Anticancer Drugs* **2011**, *22*, 763 - 773. DOI: 10.1097/CAD.0b013e328347181a.
6. Wang, Y.; Zhou, Y.; Zhou, H.; Jia, G.; Liu, J.; Han, B.; Cheng, Z.; Jiang, H.; Pan, S.; Sun, B. Pristimerin causes G1 arrest, induces apoptosis, and enhances the chemosensitivity to gemcitabine in pancreatic cancer cells. *PloS One* **2012**, *7*, e43826. DOI: 10.1371/journal.pone.0043826.
7. Deeb, D.; Gao, X.; Liu, Y.B.; Pindolia, K.; Gautam, S.C. Pristimerin, a quinonemethide triterpenoid, induces apoptosis in pancreatic cancer cells through the inhibition of pro-survival Akt/NF-κB/mTOR signaling proteins and anti-apoptotic Bcl-2. *Int J Oncol* **2014**, *44*, 1707 - 1715. DOI: 10.3892/ijo.2014.2325.
8. Liu, Y.B.; Gao, X.; Deeb, D.; Arbab, A.S.; Gautam, S.C. Pristimerin induces apoptosis in prostate cancer cells by down-regulating Bcl-2 through ROS-dependent ubiquitin-proteasomal degradation pathway. *J Carcinog Mutagen* **2013**, Suppl 6:005.
9. Huang, S.; He, P.; Peng, X.; Li, J.; Xu, D.; Tang, Y. Pristimerin inhibits prostate cancer bone metastasis by targeting PC-3 stem cell characteristics and VEGF-induced vasculogenesis of BM-EPCs. *Cell Physiol Biochem* **2015**, *37*, 253 - 268. DOI: 10.1159/000430350.
10. Lee, J.S.; Yoon, I.S.; Lee, M.S.; Cha, E.Y.; Thuong, P.T.; Diep, T.T.; Kim, J.R. Anticancer activity of pristimerin in epidermal growth factor receptor 2-positive SKBR3 human breast cancer cells. *Biol Pharm Bull* **2013**, *36*, 316 - 325.
11. Cevatemre, B.; Erkısa, M.; Aztopal, N.; Karakas, D.; Alper, P.; Tsimplouli, C.; Sereti, E.; Dimas, K.; Armutak, E.L.L.; Gurevin, E.G.; et al. A promising natural product, pristimerin, results in cytotoxicity against breast cancer stem cells *in vitro* and xenografts *in vivo* through apoptosis and an incomplete autopaghy in breast cancer. *Pharmacol Res* **2018**, *129*, 500 - 514. DOI: 10.1016/j.phrs.2017.11.027.
12. Kim, H.J.; Park, G.M.; Kim, J.K. Anti-inflammatory effect of pristimerin on lipopolysaccharide-induced inflammatory responses in murine macrophages. *Arch Pharm Res* **2013**, *36*, 495 - 500. DOI: 10.1007/s12272-013-0054-1.

13. Tong, L.; Nanjundaiah, S.M.; Venkatesha, S.H.; Astry, B.; Yu, H.; Moudgil, K.D. Pristimerin, a naturally occurring triterpenoid, protects against autoimmune arthritis by modulating the cellular and soluble immune mediators of inflammation and tissue damage. *Clin Immunol* **2014**, *155*, 220 - 230. DOI: 10.1016/j.clim.2014.09.014.
14. Jin, Y.; Wang, Y.; Zhao, D.; Ma, S.; Lu, J.; Shuang, G. Pristimerin attenuates ovalbumin-induced allergic airway inflammation in mice. *Immunopharmacol Immunotoxicol* **2016**, *38*, 221 - 227. DOI: 10.3109/08923973.2016.1168435.
15. Murayama, T.; Eizuru, Y.; Yamada, R.; Sadanari, H.; Matsubara, K.; Rukung, G.; Tolo, F.M.; Mungai, G.M.; Kofi-Tsekpo, M. Anticytomegalovirus activity of pristimerin, a triterpenoid quinone methide isolated from *Maytenus heterophylla* (Eckl. & Zeyh.) *Antivir Chem Chemother* **2007**, *18*, 133 - 139.
16. Ryu, Y.B.; Park, S.J.; Kim, Y.M.; Lee, J.Y.; Seo, W.D.; Chang, J.S.; Park, K.H.; Rho, M.C.; Lee, W.S. SARS-CoV 3CLpro inhibitory effects of quinone-methide triterpenes from *Tripterygium regelii*. *Bioorg Med Chem Lett* **2010**, *20*, 1873 - 1876. DOI: 10.1016/j.bmcl.2010.01.152.
17. Ferramosca, A.; Conte, A.; Guerra, F.; Felline, S.; Rimoli, M.G; Mollo, E.; Zara, V.; Terlizzi, A. Metabolites from invasive pests inhibit mitochondrial complex II: A potential strategy for the treatment of human ovarian carcinoma? *Biochem Biophys Res Commun* **2016**, *473*, 1133 - 1138. DOI: 10.1016/j.bbrc.2016.04.028.
18. Yu, H.; Zhang, H.; Dong, M.; Wu, Z.; Shen, Z.; Xie, Y.; Kong, Z.; Dai, X.; Xu, B. Metabolic reprogramming and AMPKα1 pathway activation by caulerpin in colorectal cancer cells. *Int J Oncol* **2017**, *50*, 161 - 172. DOI: 10.3892/ijo.2016.3794.
19. Lucena, A.M.M.; Souza, C.R.M.; Jales, J.T.; Guedes, P.M.M.; de Miranda, G.E.C.; de Moura, A.M.A.; Araújo-Júnior, J.X.; Nascimento, G.J.; Scortecci, K.C.; Santos, B.V.O.; et al. The bisindole alkaloid caulerpin, from seaweeds of the genus *Caulerpa*, attenuated colon damage in murine colitis model. *Mar Drugs* **2018**, *16*, 318. DOI: 10.3390/md16090318.
20. de Souza, E.T.; de Lira, D.P.; de Queiroz, A.C.; da Silva, D.J.; de Aquino, A.B.; Mella, E.A.; Lorenzo, V.P.; de Miranda, G.E.; de Araújo-Júnior, J.X.; Chaves, M.C.; et al. The antinociceptive and anti-inflammatory activities of caulerpin, a bisindole alkaloid isolated from seaweeds of the genus *Caulerpa*. *Mar Drugs* **2009**, *7*, 689 - 704. DOI: 10.3390/md7040689.
21. Cavalcante-Silva, L.H.; Falcão, M.A.; Vieira, A.C.; Viana, M.D.; de Araújo-Júnior, J.X.; Sousa, J.C.; da Silva, T.M.; Barbosa-Filho, J.M.; Noël, F.; de Miranda, G.E.; et al. Assessment of mechanisms involved in antinociception produced by the alkaloid caulerpine. *Molecules* **2014**, *19*, 14699 - 14709. DOI: 10.3390/molecules190914699.
22. Chay, C.I.C.; Cansino, R.G.; Pinzón, C.I.E.; Torres-Ochoa, R.O.; Martínez, R. Synthesis and anti-tuberculosis activity of the marine natural product caulerpin and its analogues. *Mar Drugs* **2014**, *12*, 1757 - 1772. DOI: 10.3390/md12041757
23. Chen, J.; Li, H.; Zhao, Z.; Xia, X.; Li, B.; Zhang, J.; Yan, X. Diterpenes from the marine algae of the genus *Dictyota*. *Mar Drugs* **2018**, *16*, 159. DOI: 10.3390/md16050159.
24. Boo, K.S.; Jung, C.H. Field tests of synthetic sex pheromone of the apple Leafminer moth, *Phyllonorycter ringoniella*. *Journal of Chemical Ecology* **1998**, *24*, 1939 - 1947.
25. Satil, F.; Azcan, N.; Baser, K.H.C. Fatty acid composition of pistachio nuts in Turkey. *Chemistry of Natural Compounds* **2003**, *39*, 322 - 324.
26. Ng, J.C.; Qi, L.; Moore, M.R. HPLC measurement of harderoporphyrin in the harderian glands of rodents as a biomarker for sub-lethal or chronic arsenic exposure. *Toxicol Lett* **2002**, *133*, 93 - 101.
27. Miranda, N.; Gerola, A.P.; Novello, C.R.; Ueda-Nakamura, T.; de Oliveira Silva, S.; Dias-Filho, B.P.; Hioka, N.; de Mello, J.C.P.; Nakamura, C.V. Pheophorbide a, a compound

isolated from the leaves of *Arrabidaea chica*, induces photodynamic inactivation of *Trypanosoma cruzi*. *Photodiagnosis Photodyn Ther* **2017**, *19*, 256 - 265. DOI: 10.1016/j.pdpdt.2017.05.004.
28. Ratnoglik, S.L.; Aoki, C.; Sudarmono, P.; Komoto, M.; Deng, L.; Shoji, I.; Fuchino, H.; Kawahara, N.; Hotta, H. Antiviral activity of extracts from *Morinda citrifolia* leaves and chlorophyll catabolites, pheophorbide a and pyropheophorbide a, against hepatitis C virus. *Microbiol Immunol* **2014**, *58*, 188 - 194. DOI: 10.1111/1348-0421.12133.
29. Chan, J.Y.; Tang, P.M.; Hon, P.M.; Au, S.W.; Tsui, S.K.; Waye, M.M.; Kong, S.K.; Mak, T.C.; Fung, K.P. Pheophorbide a, a major antitumor component purified from *Scutellaria barbata*, induces apoptosis in human hepatocellular carcinoma cells. *Planta Med* **2006**, *72*, 28 - 33.
30. Egeland, E.S.; Liaaen-Jensen, S. Ten minor carotenoids from Prasinophyceae (chlorophyta). *Phytochemistry* **1995**, *40*, 515 - 520.
31. Tsushima, M.; Matsuno, T. Carotenoid composition and two new 4-ketolutein isomers in the integuments of the red tilefish *Branchiostegus japonicus*. *Fisheries Science* **1998**, *64*, 464 - 468. DOI: 10.2331/fishsci.64.464
32. Rodriguez, G.; Schloemer, G.; Diaz, A. Use of 4-ketolutein as a food additive for egg yolk coloration. U. S. Patent US 8,337,926 B2, December 25, 2012.
33. Frank, H.A.; Cua, A.; Chynwat, V.; Young, A.; Gosztola, D.; Wasielewski, M.R. The lifetimes and energies of the first excited singlet states of diadinoxanthin and diatoxanthin: The role of these molecules in excess energy dissipation in algae. *Biochim Biophys Acta* **1996**, *1277*, 243 - 252.
34. Konishi, I.; Hosokawa, M.; Sashima, T.; Maoka, T.; Miyashita, K. Suppressive effects of alloxanthin and diatoxanthin from *Halocynthia roretzi* on LPS-induced expression of pro-inflammatory genes in RAW264. 7 cells. *J Oleo Sci* **2008**, *57*, 181 - 189.
35. Miki, W.; Yamaguchi, K.; Konosu, S. Comparison of carotenoids in the ovaries of marine fish and shellfish. *Comp Biochem Physiol B* **1982**, *71*, 7 - 11.
36. McDonough, L.M.; Hoffmann, M.P.; Bierlleonhardt, B.A.; Smithhisler, C.L.; Bailey, J.B., Davis, H.G. Sex pheromone of the avocado pest, *Amorbia cuneana* (Walsingham) (Lepidoptera: Tortricidae): Structure and synthesis. *J Chem Ecol* **1982**, *8*, 255 - 265. DOI: 10.1007/BF00984021.
37. Wen, D.; Rivera-Perez, C.; Abdou, M.; Jia, Q.; He, Q.; Liu, X.; Zyaan, O.; Xu, J.; Bendena, W.G.; Tobe, S.S.; et al. Methyl farnesoate plays a dual role in regulating *Drosophila* metamorphosis. *PLoS Genet* **2015**, *11*, e1005038. DOI: 10.1371/journal.pgen.1005038.
38. Cardoso-Júnior, C.A.M.; Silva, R.P.; Borges, N.A.; de Carvalho, W.J. Walter, S.L.; Simões, Z.L.P.; Bitondi, M.M.G.; Ueira-Vieira C.; Bonetti, A.M.; Hartfelder, K. Methyl farnesoate epoxidase (mfe) gene expression and juvenile hormone titers in the life cycle of a highly eusocial stingless bee, *Melipona scutellaris*. *J Insect Physiol* **2017**, *101*, 185 - 194. DOI: 10.1016/j.jinsphys.2017.08.001.

44*.Nagaraj, S.R.; Osborne, J.W. Bioactive compounds from *Caulerpa racemosa* as a potent larvicidal and antibacterial agent. *Front. Biol.* **2014**, *9*, 300 – 305. DOI: 10.1007/s11515-014-1312-4
62*. Gao, X.; Liu, Y.; Deeb, D.; Arbab, A.S.; Gautam, S.C. Anticancer activity of pristimerin in ovarian carcinoma cells is mediated through the inhibition of prosurvival Akt/NF-κB/mTOR signaling. *J Exp Ther Oncol* **2014**, *10*, 275 – 283.
63*. Sheng, Q.; Fang, X.; Zhu, Z.; Xiao, W.; Wang, Z.; Ding, G.; Zhao, L.; Li, Y.; Yu, P.; Ding, Z.; et al. Seasonal variation of pheophorbide a and flavonoid in different organs of two *Carpinus* species and its correlation with immunosuppressive activity. *In Vitro Cell Dev Biol Anim* **2016**, *52*, 654 - 661. DOI: 10.1007/s11626-016-0041-1.

64*. Stermitz, F.R.; Tawara-Matsuda, J.; Lorenz, P.; Mueller, P.; Zenewicz, L.; Lewis, K. 5'-Methoxyhydnocarpin-D and Pheophorbide A: *Berberis* species components that potentiate berberine growth inhibition of resistant *Staphylococcus aureus*. *J Nat Prod* **2000**, *63*, 1146 - 1149.
*references are cited in main text.

Supplementary Material Table S2: Molecular Formula of 25 Unknown Compounds from *C. racemosa* Chloroform Extract.

No.	Formula	Mass	m/z
1	$C_4 H_{12} O_2 S_2$	156.02781	157.03509
2	$C_{21} H_{40} O S$	340.28029	341.28732
3	$C_{23} H_{16} N_2 O_4$	384.11126	385.11829
4	$C_{22} H_{25} N_5 O_2$	391.20081	392.20754
5	$C_{14} H_{33} N_9 O S$	375.25339	393.28682
6	$C_{28} H_{49} N O_6$	495.35626	496.36363
7	$C_{28} H_{51} N O_6$	497.37183	498.3793
8	$C_{28} H_{53} N O_6$	499.38752	500.39495
9	$C_{24} H_{49} N_{11} O S$	539.38349	540.39043
10	$C_{33} H_{55} N O_6$	561.40325	562.41052
11	$C_{38} H_{53} N_3 O$	567.4197	568.42687
12	$C_{29} H_{53} N_5 O_7$	583.39411	584.40116
13	$C_{31} H_{53} N_9 S$	583.41502	584.42149
14	$C_{36} H_{53} N_5 O_2$	587.4194	588.4262
15	$C_{35} H_{59} N O_6$	589.43467	590.44186
16	$C_{38} H_{53} N_3 O_3$	599.40895	600.41655
17	$C_{39} H_{51} N_7$	617.4208	618.42806
18	$C_{14} H_3 N O_4 S_2$	312.95072	311.94352
19	$C_{25} H_{14} N_6 S$	430.10007	429.09259
20	$C_{24} H_{27} N O_3 S_2$	441.14341	440.13623
21	$C_{26} H_{27} N O_3 S_2$	465.14329	464.1363
22	$C_{25} H_{16} N_6 O_2 S_2$	496.07662	495.06912
23	$C_{15} H_8 N_4 O_8 S_4$	499.92316	498.9159
24	$C_{20} H_{46} N_{10} O_5 S$	538.33685	537.32925
25	$C_{36} H_6 O S_5$	613.90261	612.89583

Supplementary Materials Figures S1 & S2: Chromatograms

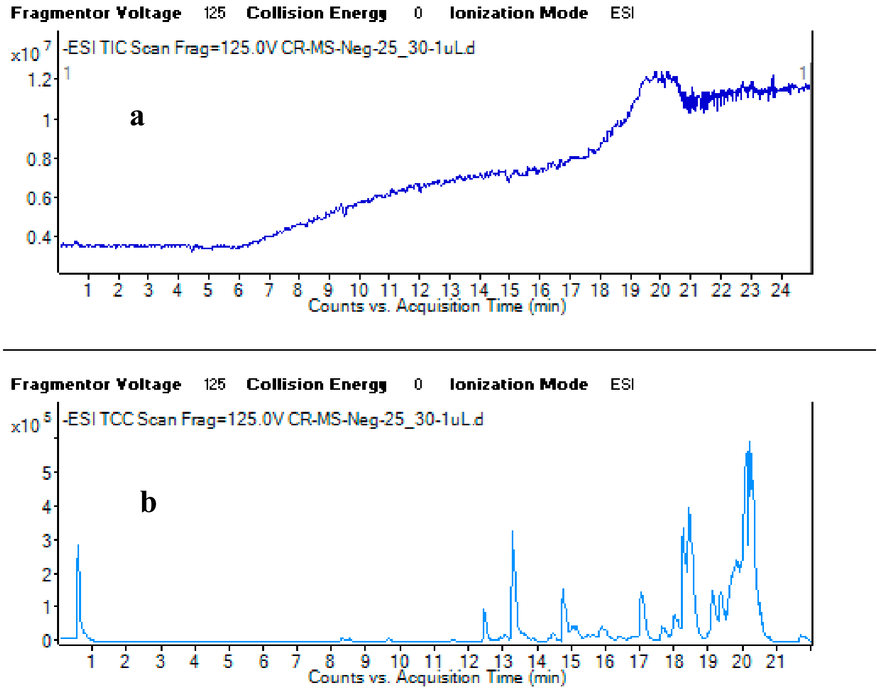

Supplementary Material Figure S1: *C. racemosa* chloroform extract was subjected to LCMS qualitative analysis using negative ion mode. The compounds were separated based on m/z ratio and retention time in the column, a) showing total ion current (TIC) chromatogram and b) showing total compound chromatogram (TCC). 48 peaks are observed in the extract.

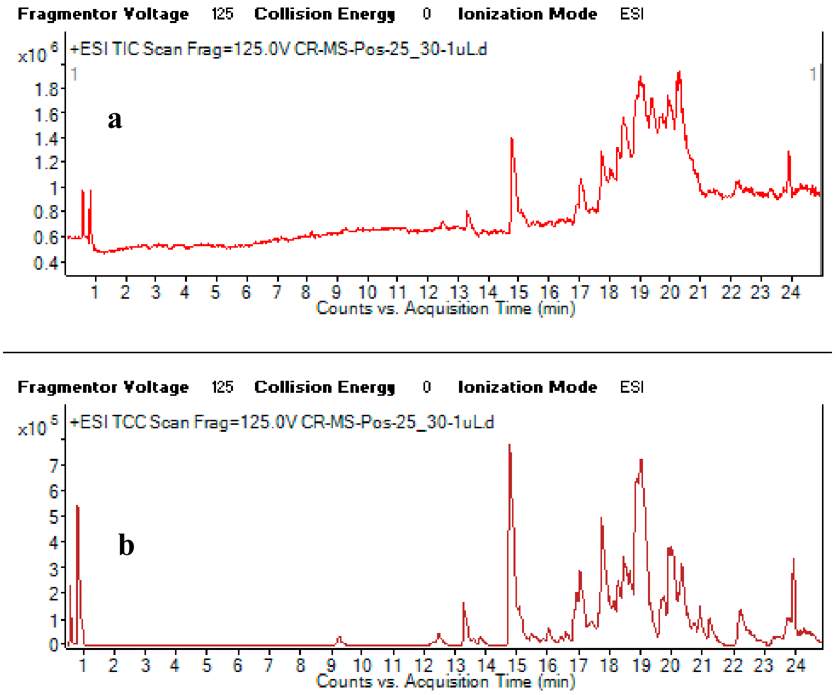

Supplementary Material Figure S2: *C. racemosa* chloroform extract was subjected to LCMS qualitative analysis using positive ion mode. The compounds were separated based on m/z ratio and retention time in the column, a) showing total ion current (TIC) chromatogram and b) showing total compound chromatogram (TCC). 74 peaks are observed in the extract.

Supplementary Material Figure S3: Mass spectra of individual compounds listed in Supplementary Material Table S1.

Compound 1: Pristimerin

Compound Label	Name	m/z	RT	Algorithm	Mass
Cpd 31: Pristimerin; C30 H40 O4; 15.904	Pristimerin	509.29092	15.9	Find by Molecular Feature	464.29287

Compound Chromatograms

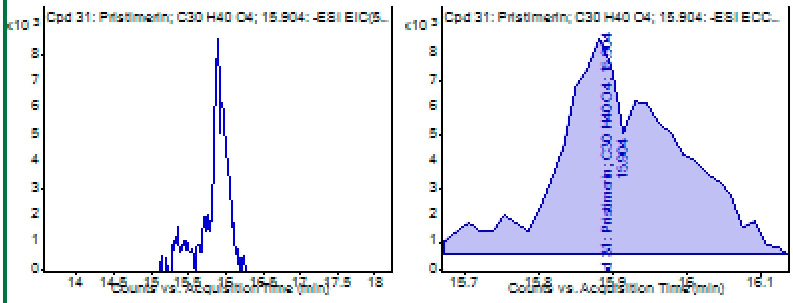

MFE MS Spectrum

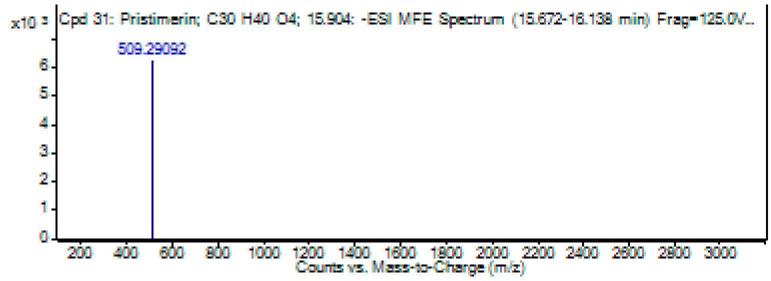

Compound 2: Caulerpin

Compound Label	Name	m/z	RT	Algorithm	Mass
Cpd 25: Caulerpin; C24 H18 N2 O4; 14.762	Caulerpin	397.12012	14.76	Find by Molecular Feature	398.12732

Compound Chromatograms

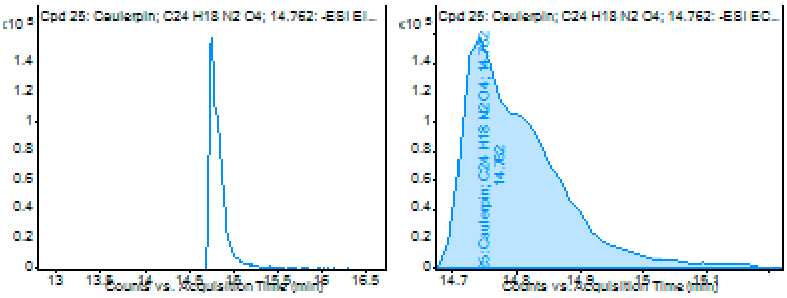

MFE MS Spectrum

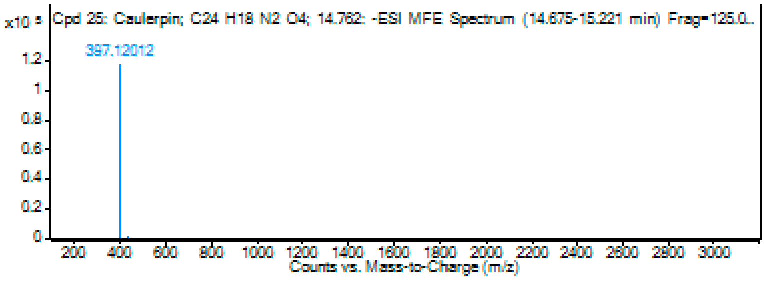

Compound 3: 5(S)-HETE lactone

Compound Label	Name	m/z	RT	Algorithm	Mass
Cpd 45: 5(S)-HETE lactone; C20 H30 O2; 18.280	**5(S)-HETE lactone**	301.21792	18.28	Find by Molecular Feature	302.22512

Compound Chromatograms

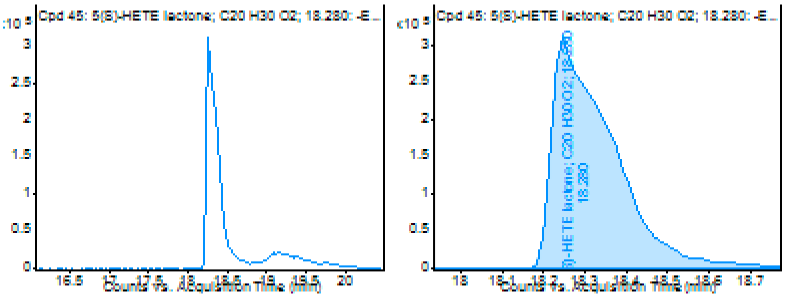

MFE MS Spectrum

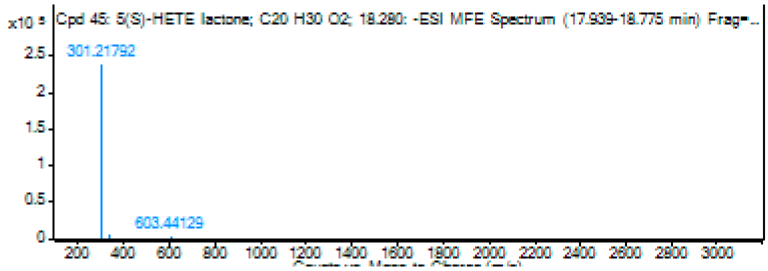

Compound 4: Isoamijiol

Compound Label	Name	m/z	RT	Algorithm	Mass
Cpd 52: (-)-Isoamijiol; C20 H32 O2; 19.127	**(-)-Isoamijiol**	303.2334	19.13	Find by Molecular Feature	304.24063

Compound Chromatograms

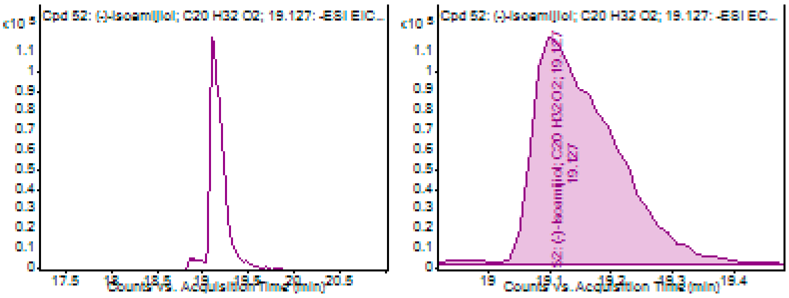

MFE MS Spectrum

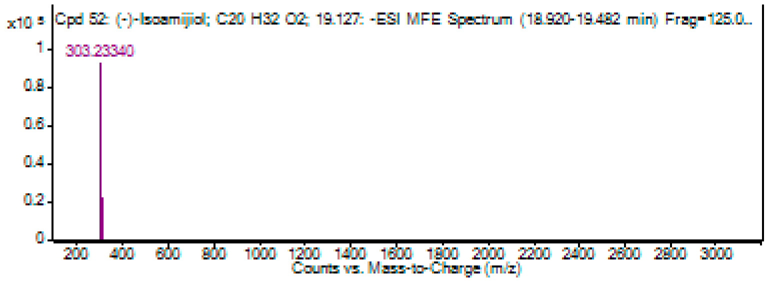

Compound 5: 12-oxo-10Z-octadecenoic acid

Compound Label	Name	m/z	RT	Algorithm	Mass
Cpd 30: 12-oxo-10Z-octadecenoic acid; C18 H32 O3; 15.854	**12-oxo-10Z-octadecenoic acid**	295.2282	15.85	Find by Molecular Feature	296.2353

Compound Chromatograms

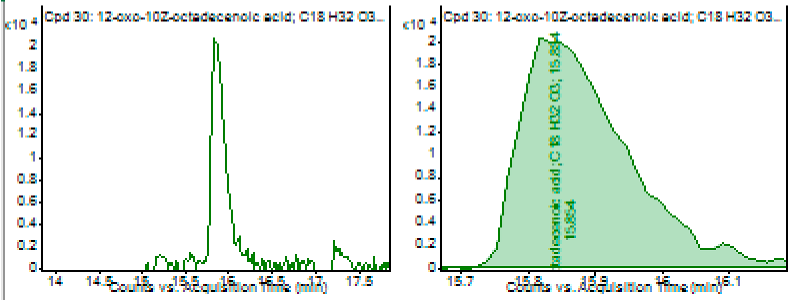

MFE MS Spectrum

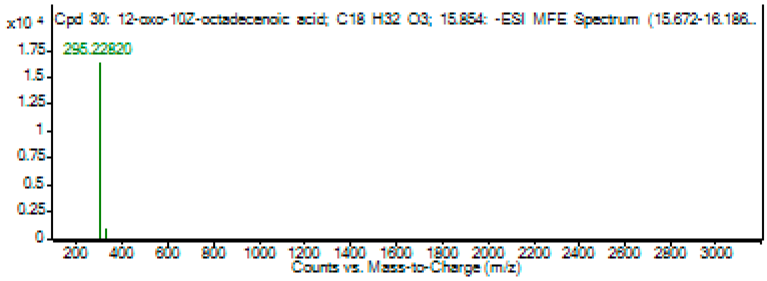

Compound 6: 12(13)epoxy-9Z,15Z-octadecadienoic acid

Compound Label	Name	m/z	RT	Algorithm	Mass
Cpd 26: α-9(10)-EpODE; C18 H30 O3; 15.075	**α-9(10)-EpODE**	293.21222	15.08	Find by Molecular Feature	294.21965

Compound Chromatograms

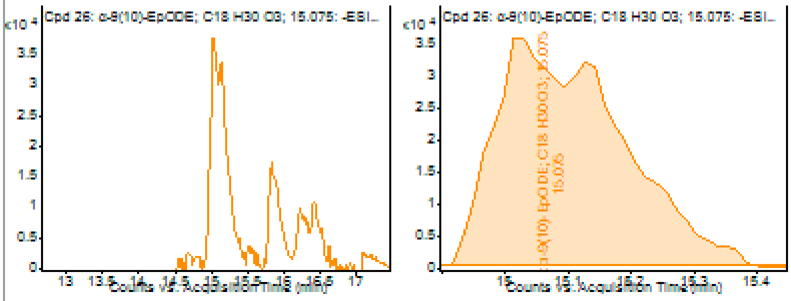

MFE MS Spectrum

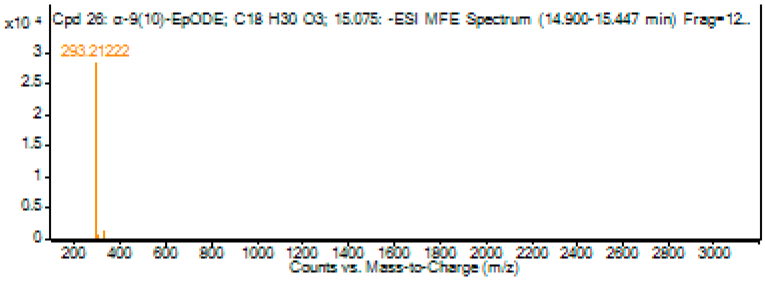

Compound 7: 9Z, 12Z, 15E-octadecatrienoic acid

Compound Label	Name	m/z	RT	Algorithm	Mass
Cpd 20: 9Z,12Z,15E-octadecatrienoic acid; C18 H30 O2; 15.855	**9Z,12Z,15E-octadecatrienoic acid**	279.23125	15.86	Find by Molecular Feature	278.22405

Compound Chromatograms

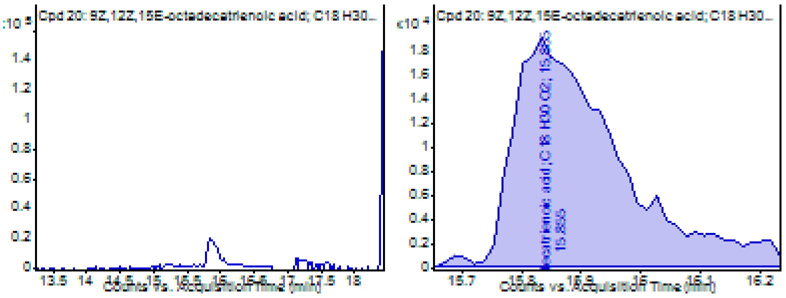

MFE MS Spectrum

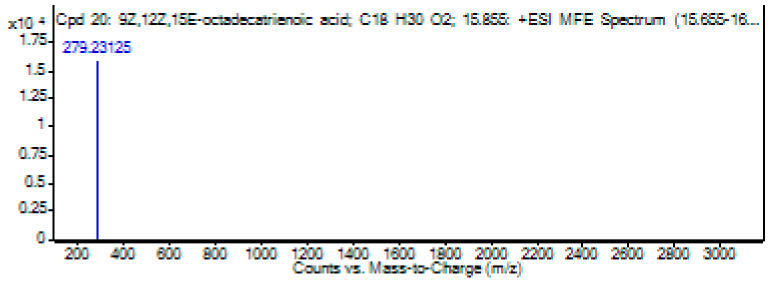

Compound 8: 4E, 10Z-tetradecadienyl acetate

Compound Label	Name	m/z	RT	Algorithm	Mass
Cpd 38: 10E,12Z-Tetradecadienyl acetate; C16 H28 O2; 18.026	**10E,12Z-Tetradecadienyl acetate**	253.21626	18.03	Find by Molecular Feature	252.20921

Compound Chromatograms

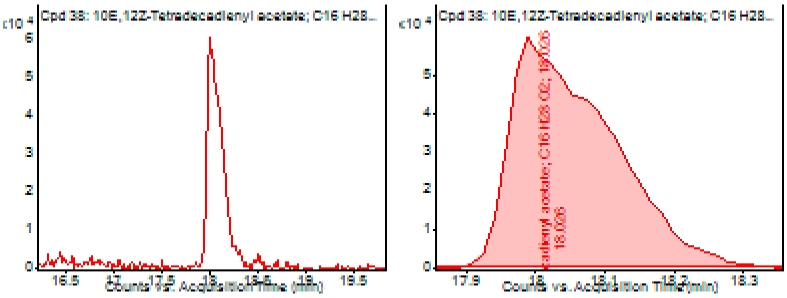

MFE MS Spectrum

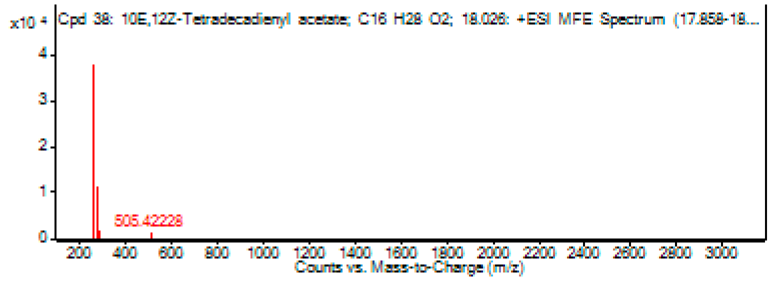

Compound 9: trans-5, trans-8, trans-11, hexadecatrioenoic acid

Compound Label	Name	m/z	RT	Algorithm	Mass
Cpd 37: trans-5, trans-8, trans-11-hexadecatrienoic acid; C16:3n-5,8,11; C16 H26 O2; 17.046	**trans-5, trans-8, trans-11-hexadecatrienoic acid; C16:3n-5,8,11**	249.18627	17.05	Find by Molecular Feature	250.19348

Compound Chromatograms

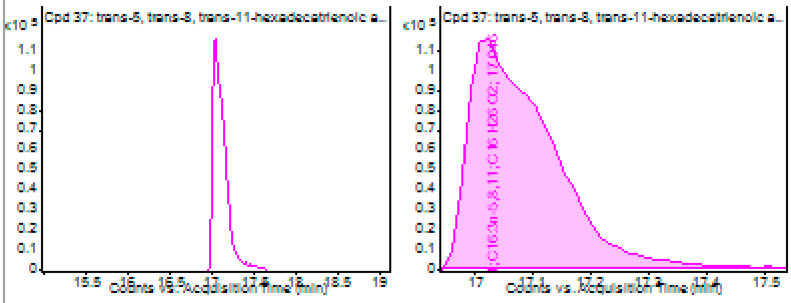

MFE MS Spectrum

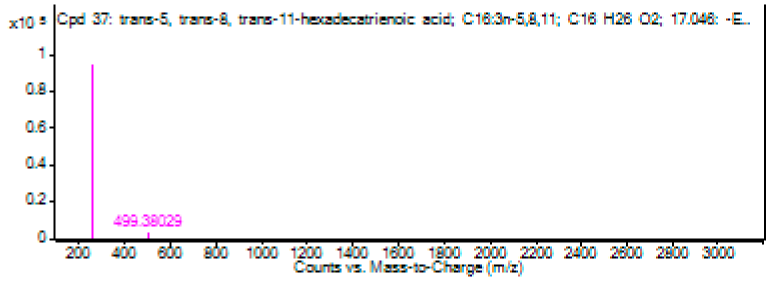

Compound 10: 7-octadecenoic acid

Compound Label	Name	m/z	RT	Algorithm	Mass
Cpd 55: 7Z,10Z-octadecadienoic acid; C18 H32 O2; 19.372	**7Z,10Z-octadecadienoic acid**	279.23323	19.37	Find by Molecular Feature	280.24054

Compound Chromatograms

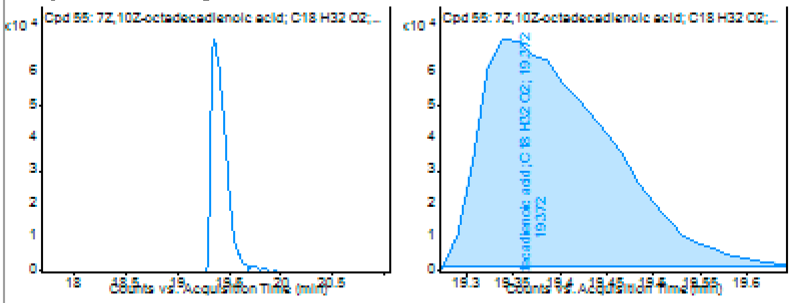

MFE MS Spectrum

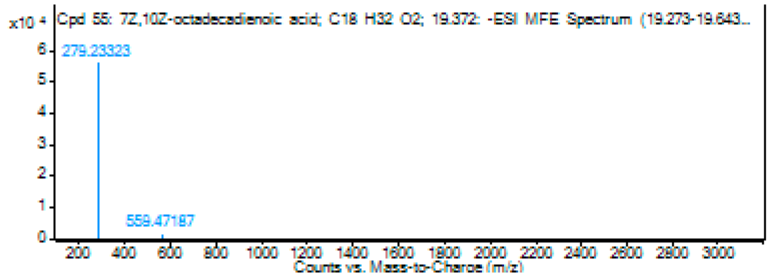

Compound 11: Harderoporphyrin

**Compound Label**	**Name**	**m/z**	**RT**	**Algorithm**	**Mass**
Cpd 61: Harderoporphyrin; C35 H36 N4 O6; 19.624	**Harderoporphyrin**	609.27131	19.62	Find by Molecular Feature	608.26415

Compound Chromatograms

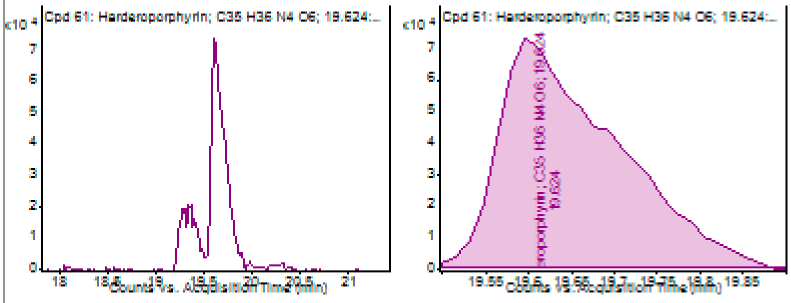

MFE MS Spectrum

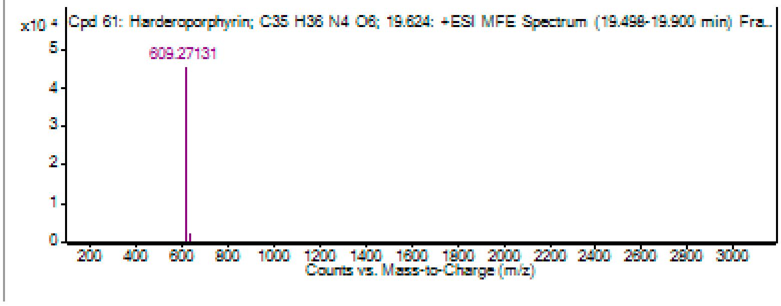

Compound 12: Pheophorbide a

**Compound Label**	**Name**	**m/z**	**RT**	**Algorithm**	**Mass**
Cpd 66: Pheophorbide a; C35 H36 N4 O5; 19.922	**Pheophorbide a**	593.27678	19.92	Find by Molecular Feature	592.26945

Compound Chromatograms

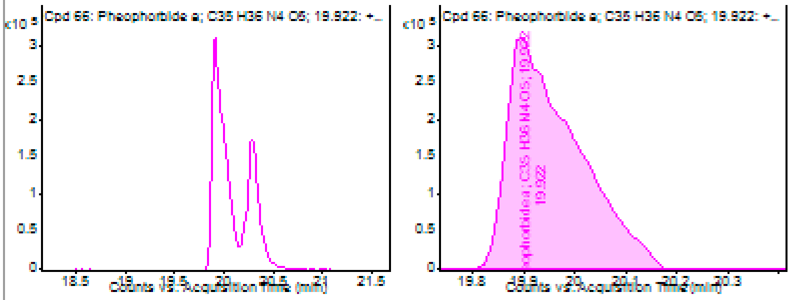

MFE MS Spectrum

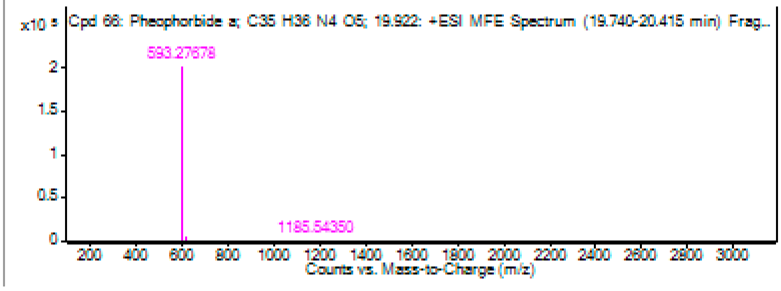

Compound 13: Micromonal

Compound Label	Name	m/z	RT	Algorithm	Mass
Cpd 63: Micromonal; C40 H56 O3; 19.698	**Micromonal**	585.42951	19.7	Find by Molecular Feature	584.42184

Compound Chromatograms

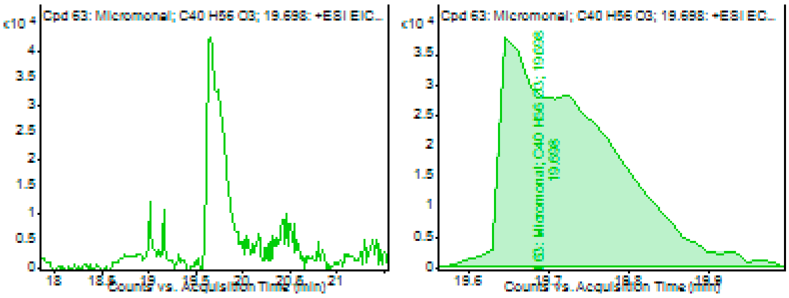

MFE MS Spectrum

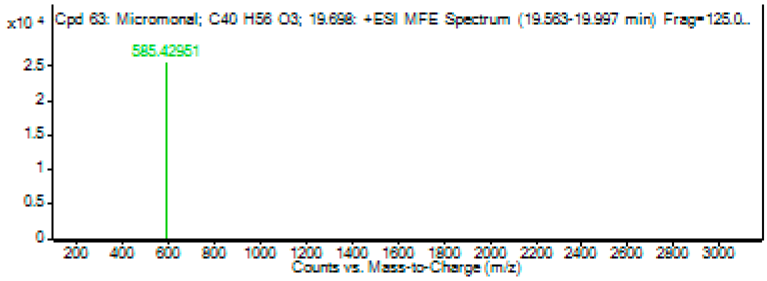

Compound 14: 4-ketolutein D and 4-ketolutein F

Compound Label	Name	m/z	RT	Algorithm	Mass
Cpd 35: 4-Ketolutein D; C40 H54 O3; 17.756	**4-Ketolutein D**	583.41537	17.76	Find by Molecular Feature	582.40796

Compound Chromatograms

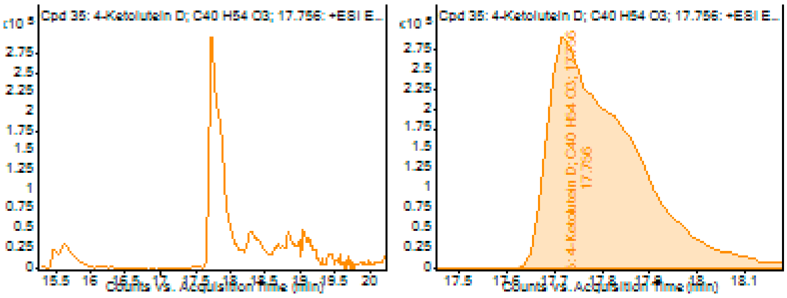

MFE MS Spectrum

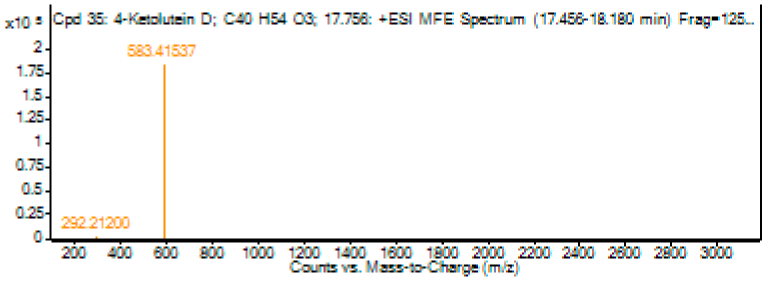

Compound Label	Name	m/z	RT	Algorithm	Mass
Cpd 48: 4-Ketolutein F; C40 H54 O3; 18.838	**4-Ketolutein F**	583.41452	18.84	Find by Molecular Feature	582.40748

Compound Chromatograms

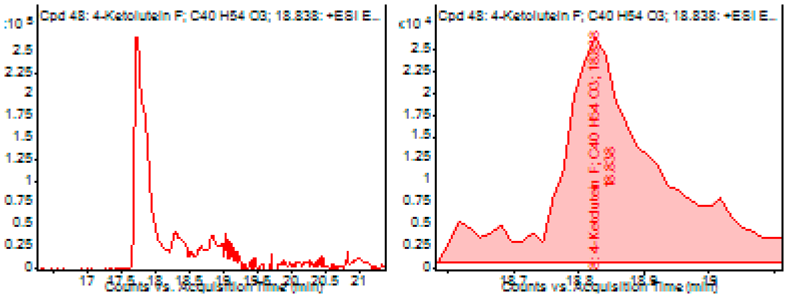

MFE MS Spectrum

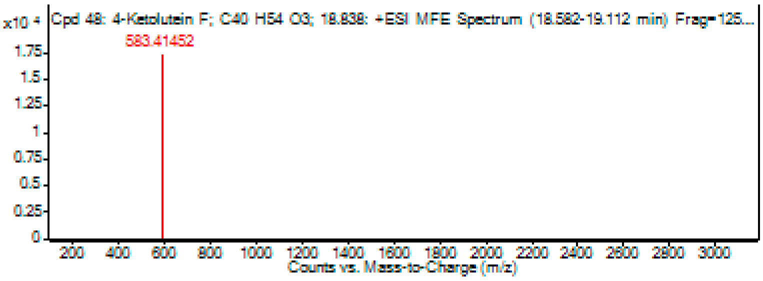

Compound 15: Diatoxanthin/7,8-Didehydrozeaxanthin

Compound Label	Name	m/z	RT	Algorithm	Mass
Cpd 62: Diatoxanthin/ 7,8-Didehydrozeaxanthin; C40 H54 O2; 19.679	**Diatoxanthin/ 7,8-Didehydrozeaxanthin**	567.41984	19.68	Find by Molecular Feature	566.4117

Compound Chromatograms

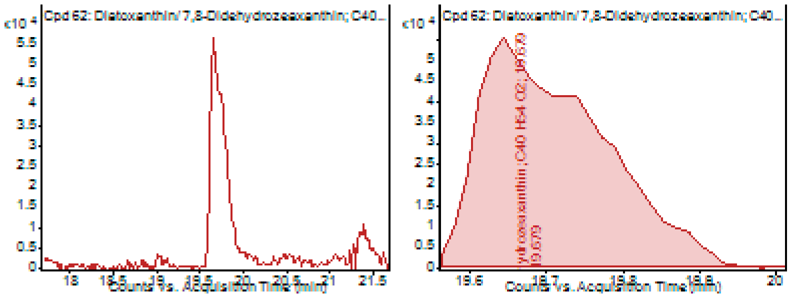

MFE MS Spectrum

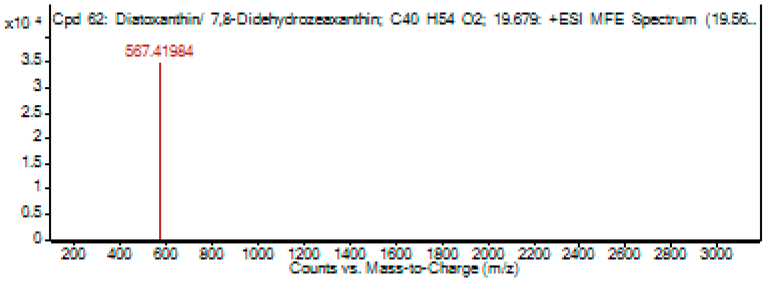

Compound 16: Pectenoxanthin; Alloxanthin; Manixanthin

Compound Label	Name	m/z	RT	Algorithm	Mass
Cpd 36: Alloxanthin/ Tetradehydrozeaxanthin / (Cynthiaxanthin)/ (Pectenoxanthin); C40 H52 O2; 17.756	**Alloxanthin/ Tetradehydrozeaxanthin / (Cynthiaxanthin)/ (Pectenoxanthin)**	565.40454	17.76	Find by Molecular Feature	564.39704

Compound Chromatograms

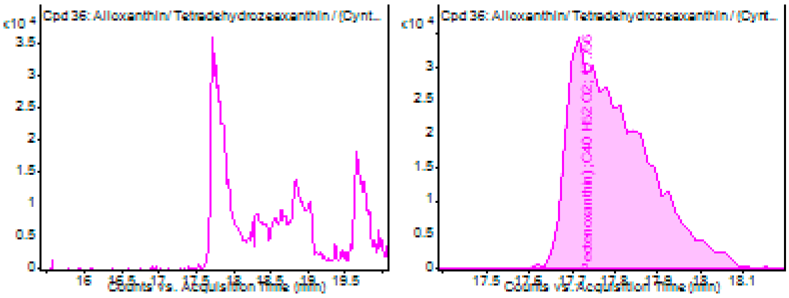

MFE MS Spectrum

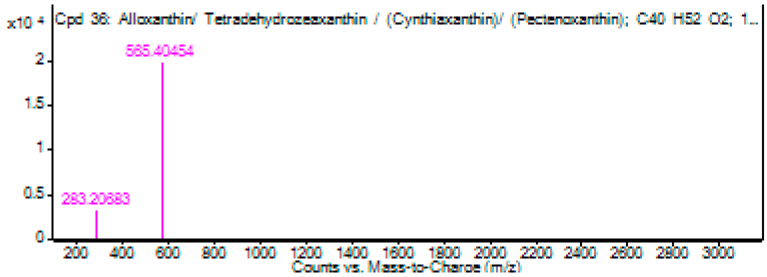

Compound 17: 1α-hydroxy-24-methylvitamin D2/1α-hydroxy-24-methylergocalciferol

Compound Label	Name	m/z	RT	Algorithm	Mass
Cpd 65: 1α-hydroxy-24-methylvitamin D2 / 1α-hydroxy-24-methylergocalciferol; C29 H46 O2; 19.769	**1α-hydroxy-24-methylvitamin D2 / 1α-hydroxy-24-methylergocalciferol**	427.35738	19.77	Find by Molecular Feature	426.34966

Compound Chromatograms

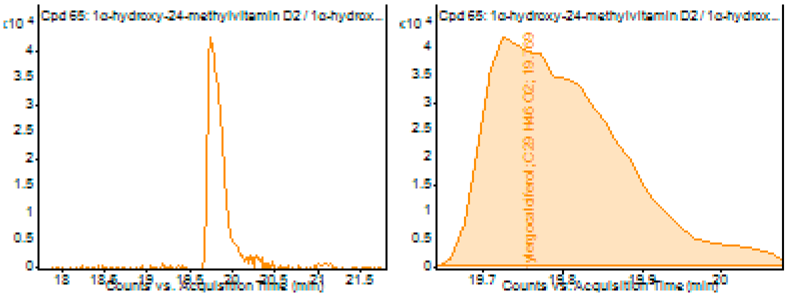

MFE MS Spectrum

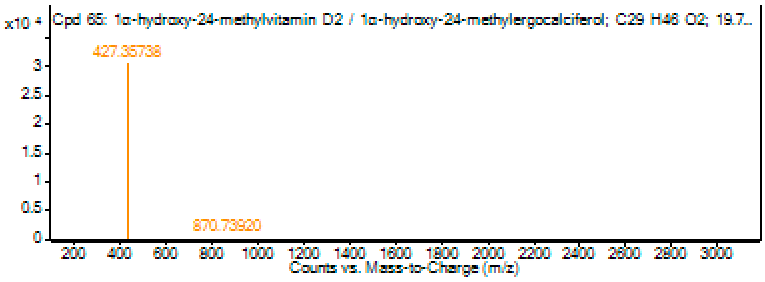

Compound 18: Coprocholic acid; 3α,12α-Dihydroxy-5β-chol-8(14)-en-24-oic Acid

Compound Label	Name	m/z	RT	Algorithm	Mass
Cpd 44: 3α,12α-Dihydroxy-5β-chol-8(14)-en-24-oic Acid; C24 H38 O4; 18.448	**3α,12α-Dihydroxy-5β-chol-8(14)-en-24-oic Acid**	391.28476	18.45	Find by Molecular Feature	390.27804

Compound Chromatograms

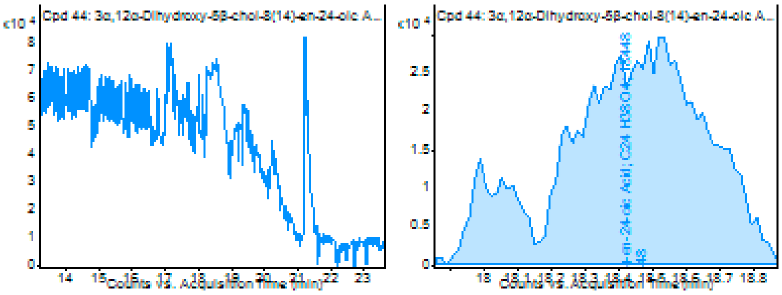

MFE MS Spectrum

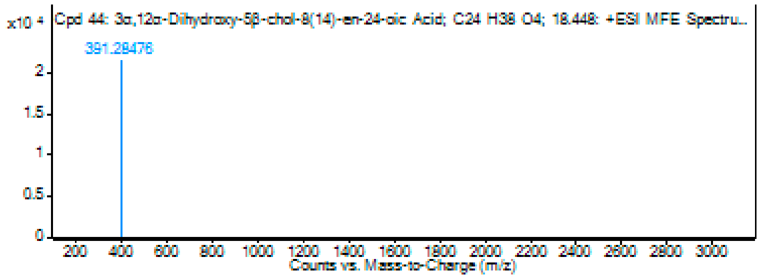

Compound 19: 9Z, 11Z, 13E, 15E-Octadecatetraenoic acid

Compound Label	Name	m/z	RT	Algorithm	Mass
Cpd 15: 5Z,8Z,11Z,14Z-octadecatetraenoic acid; C18 H28 O2; 15.093	**5Z,8Z,11Z,14Z-octadecatetraenoic acid**	277.21633	15.09	Find by Molecular Feature	276.20918

Compound Chromatograms

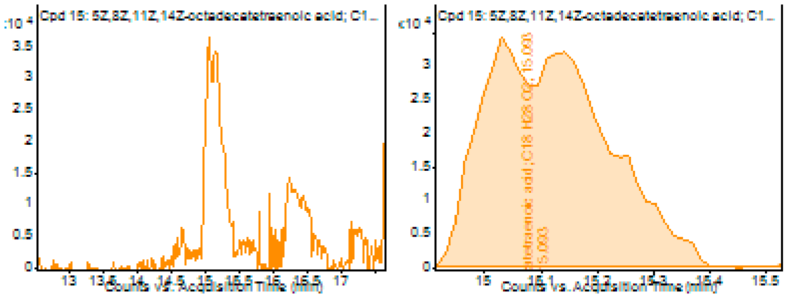

MFE MS Spectrum

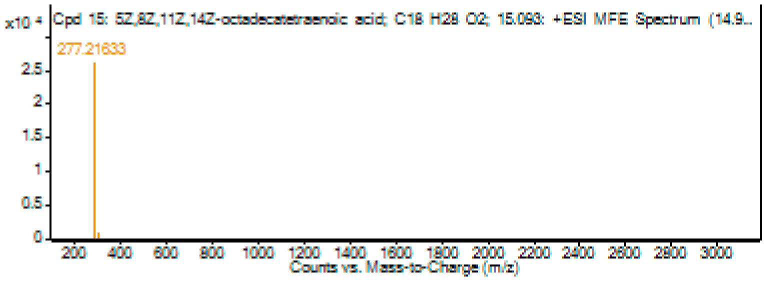

Compound 20: 9Z, 12Z, 15E-occtadecatrienoic acid

Compound Label	Name	m/z	RT	Algorithm	Mass
Cpd 20: 9Z,12Z,15E-octadecatrienoic acid; C18 H30 O2; 15.855	**9Z,12Z,15E-octadecatrienoic acid**	279.23125	15.86	Find by Molecular Feature	278.22405

Compound Chromatograms

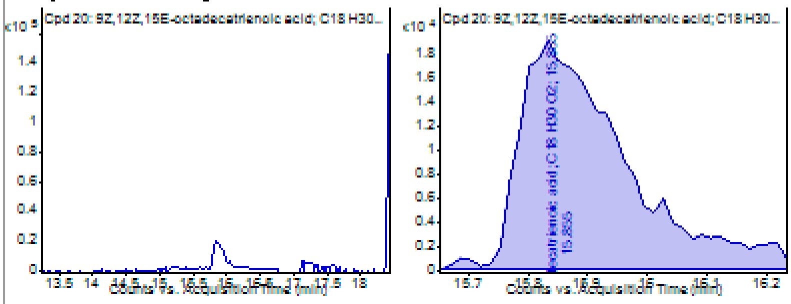

MFE MS Spectrum

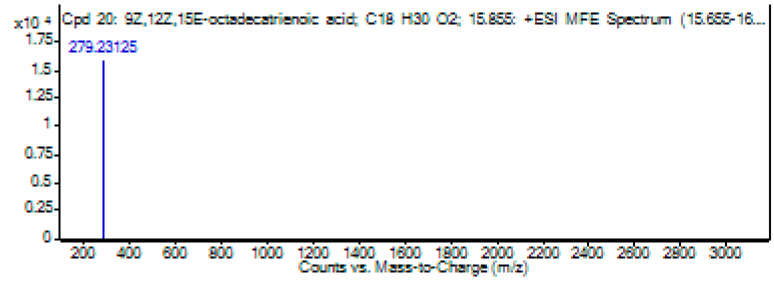

Compound 21: 5Z, 8Z, 11Z, 14Z-octadecatetraenoic acid

Compound Label	Name	m/z	RT	Algorithm	Mass
Cpd 34: 5Z,8Z,11Z,14Z-octadecatetraenoic acid; C18 H28 O2; 17.665	**5Z,8Z,11Z,14Z-octadecatetraenoic acid**	277.21651	17.67	Find by Molecular Feature	276.2094

Compound Chromatograms

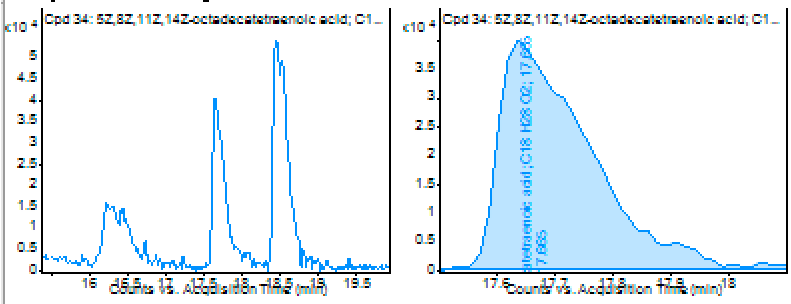

MFE MS Spectrum

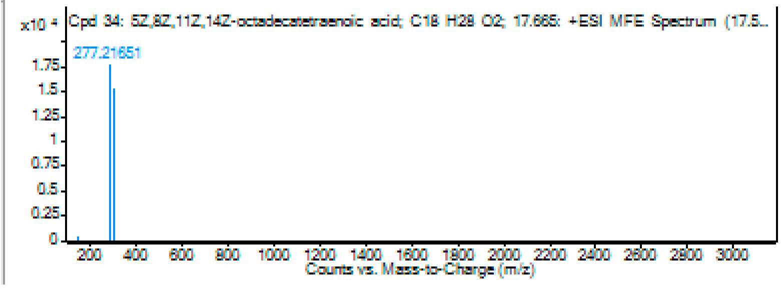

Compound 22: 10E, 12Z-tetradecadienyl acetate

Compound Label	Name	m/z	RT	Algorithm	Mass
Cpd 38: 10E,12Z-Tetradecadienyl acetate; C16 H28 O2; 18.026	**10E,12Z-Tetradecadienyl acetate**	253.21626	18.03	Find by Molecular Feature	252.20921

Compound Chromatograms

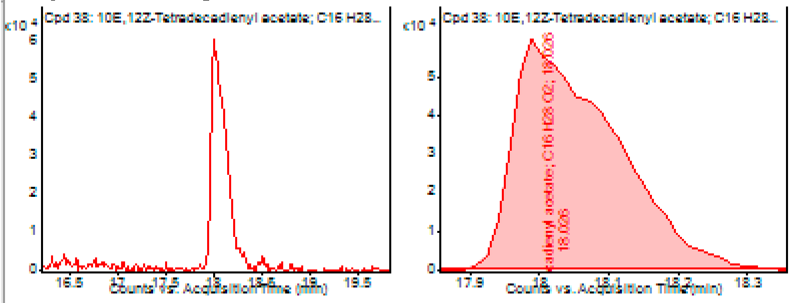

MFE MS Spectrum

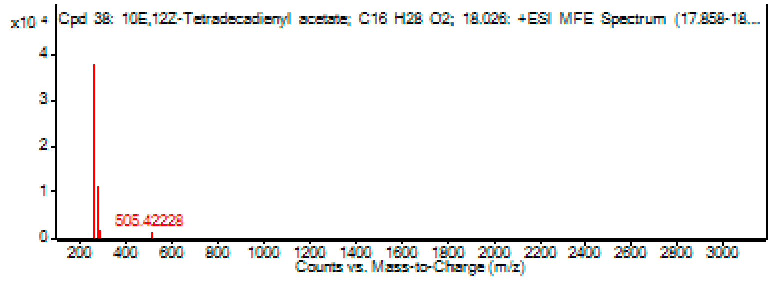

Compound 23: Methyl farnesoate

Compound Label	Name	m/z	RT	Algorithm	Mass
Cpd 27: Methyl farnesoate; C16 H26 O2; 17.058	**Methyl farnesoate**	251.2006	17.06	Find by Molecular Feature	250.19341

Compound Chromatograms

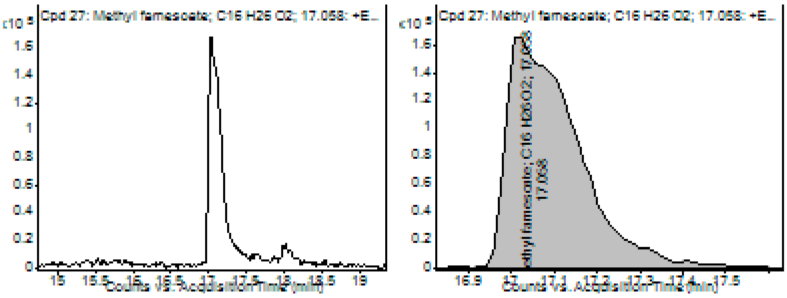

MFE MS Spectrum

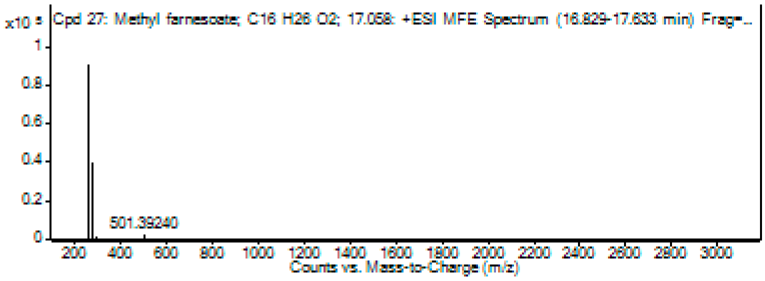

Supplementary Material Figure S3: Mass spectra of individual compounds listed in Supplementary Material Table S1.
